# Mechanisms of Barium Sulfate Dissolution through the Lens of Kinetic Monte Carlo Simulations

**DOI:** 10.1021/acsomega.5c05761

**Published:** 2025-10-10

**Authors:** Nikolai Trofimov, Andreas Luttge, Inna Kurganskaya

**Affiliations:** 1 9168 University of Bremen, FB5 Geo, Klagenfurter Str. 4, Bremen 28359, Germany; 2 Earth, Environmental, and Planetary Sciences Department, Rice University, 6100 Main St, Houston TX77005, United States

## Abstract

The prediction of crystalline matter dissolution kinetics is one of the main focuses in environmental science, civil engineering, chemical kinetics, synthesis, drug delivery, and other scientific and industrial fields. Our ability to predict the temporal dynamics of material fluxes is crucial for designing crystalline materials and controlling the behavior of chemical systems for various applications. The critical question is, do these dynamics have deterministic or stochastic features, or should we expect a constant, oscillatory, or completely random temporal behavior from a predefined crystalline structure? Our study is dedicated to barium sulfate (barite), which is considered one of the primary backfilling materials for nuclear waste repositories. We developed a new parametrized Kinetic Monte Carlo (kMC) model, which allows us to simulate the temporal evolution of the system. We have found that material flux oscillates quasiperiodically over time, indicating the presence of deterministic and stochastic components. This raises the question of whether it can be predicted in principle. Our observations cover the mechanistic and kinetic behavior of the Barite-water system and can be applied to studies of other solid–liquid interfaces.

## Graphical abstract

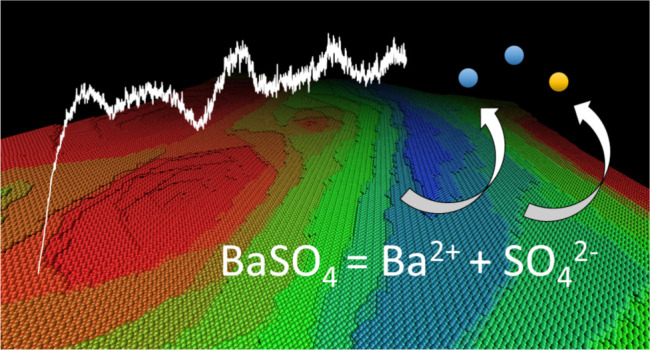

## Introduction

The barite–water system is a subject of particular interest for environmental science, geochemistry of Ba, and industrial applications. Barite is the main mineral of Ba in the Earth’s crust, widely formed in sedimentary and hydrothermal processes. Barium extracted from barite is used in various industrial applications, including the production of ceramics, glass, plastics, paints, ferrites, titanites, etc. Barite is also used in the manufacturing of fillers, extenders, and weighting agents. However, the leading industrial application is using the mineral as a weighting agent in oil and gas drilling fluids.^1^ One of the problems in the oil and gas industry is the mineralization of production tubes by poorly soluble barite (Kp = 10^–10^,^2^). This problem has been solved by the application of organic chelating agents such as EDTA and DTPA. The EDTA- and DTPA-promoted dissolution of barite was broadly studied through experimental techniques.^3–10^ One of the coproducts of oil and gas extraction is the ^226^Ra isotope. Due to the similar ionic radii of Ba and Ra, barite can incorporate ^226^Ra into the mineral’s lattice.^11–15^ According to the prognoses^16^ ^226^Ra isotope will significantly contribute to the overall dose of nuclear waste in 10^4^–10^5^ years. The ability of barite to form solid solutions with radioactive isotopes of Ra and Sr makes this mineral a promising material for the engineering of safe and reliable nuclear waste repositories.

Barite is considered as a possible backfilling and shielding material^17–19^ for nuclear waste in underground storage.^20^ The materials used in the construction of nuclear waste repositories are susceptible to corrosion. As a result, they will encounter groundwater, triggering dissolution and back-precipitation reactions. Thus, the environmental fate of Ra and Sr strongly depends on the kinetics of dissolution and back-precipitation in the backfilling matrix. Therefore, information about the dissolution mechanisms and rates of the minerals is necessary for achieving the highest levels of nuclear repository safety.

Mineral dissolution at the microscopic scale is a complex process involving the formation of reactive surface features that act as major sources of dissolved material flux. Atomic steps and etch pits are the reactive features commonly observed at mineral surfaces using microscopic techniques, e.g., atomic force microscopy (AFM)^8,9,21–29^ or VSI.^30–38^ The dissolution rate values in the material flux units (mol/cm^2^·s) can be directly obtained from time lapses of microscopically measured surface height maps, as shown in the works of Fischer et al.^39,40^ The velocity of atomic step propagation, measured in nanometers per second, is another kinetic parameter commonly measured during in situ AFM experiments. Although these methods provide robust and reliable information about the dissolution process at the microscopic scale, it remains challenging to access the mechanisms of reactive surface feature emergence from molecular-scale processes of bond breaking and detachment. The approaches of computational chemistry, such as molecular dynamics^41–43^ ^,^ ^43–49^ ^,^ ^49–51^ ^,^ ^51–56^ or ab initio,^57–63^ provide valuable insights into the electronic structure details of bond hydrolysis and the detachment of atoms from the surface.^64–67^ The kinetic Monte Carlo method^68–72^ can help us close the scale gaps and link molecular-scale details with microscopically observed kinetic behavior.

The kinetic Monte Carlo approach to studying mechanisms of mineral dissolution and growth has a three-decade history, starting with the seminal work of multiple authors (e.g., Wehrli, Blum, and Lasaga) who related the formation of etch pits to lattice defects and led us into the era of *ab initio* parametrized kMC models,^73–80^ even with the application of Machine Learning.^81–83^ Ideally, fully and correctly parametrized models can be used to quantitatively predict material fluxes and simulate expected dissolution scenarios in laboratory or natural environments. Practically, however, this is rarely the case due to the large number of individual surface reactions required to cover all possible molecular reactions that may potentially occur on the surface. Our previous publications demonstrated how reasonable arguments based on the physical chemistry of mineral-water interface structure, ab initio and MD calculations, can be combined to introduce and test parametrization work hypotheses.^84,85^ In particular, we demonstrated the importance of the second coordination sphere for the proper parametrization of models based on recognizing local molecular neighborhoods.^86,87^ In some cases, however, such as ionic crystals, the first coordination sphere neighborhood is sufficient to reproduce experimental data.^84,85^ As we have previously demonstrated, a direct comparison between simulated and experimentally obtained surface morphologies allows us to evaluate the correctness of the parametrization approach and parameter values.^86–88^ This process of comparison, followed by the evaluation of correctness, is essential for validating the model and testing the working hypothesis.

The dissolution of barite presents an interesting and challenging problem in terms of constructing a proper kMC model. Our preliminary studies^88^ showed that incorporating the second coordination sphere and parameter variation alone is not sufficient to reproduce experimentally observed dissolution surface features. Location-specific steric factors were introduced to obtain realistic surface morphologies.^88^ However, the choice of local neighborhoods for parameter corrections was relatively intuitive. Here, we introduce a new kMC model of barite dissolution based on a bond-length-dependent parametrization approach. The primary motivation here is to reflect distance-dependent Coulomb interactions that affect local bond energies and activation barriers. The crystallographic structure of barite is indeed characterized by an uneven direction-specific distribution of bond lengths, which may play a role in location-specific dissolution rates. Another model improvement is the use of temperature-dependent atomic step velocities to separate activation energy parameters from reaction attempt frequencies. We show here the results of the new parametrization approach and discuss mechanisms of barite dissolution at the microscopic scale revealed in the simulations. In addition, we demonstrate a new and kinetically unique behavior of surfaces with interacting etch pits that has not previously been reported. In particular, we demonstrate steady-period oscillations of the material flux, which are not observed in other modeled systems. This new result opens a new venue for understanding the role of crystal structure in defining principally different dynamic and statistical behaviors of dissolution rates.

## Methods

### Modeled System Setup

We performed kMC simulations using the crystallographic data on a barite unit cell.^89^ The barite structure belongs to the *Pnma* space group and has the following unit cell parameters: a = 8.884(4) Å, b = 5.458(0) Å, and c = 7.153(3) Å. The unit cell contains four Ba atoms and four SO_4_ tetrahedra connected via oxygen atoms (Figure 1). The unit cell has two half-unit cell layers inverted by the 2_1_ screw axis in the c direction. For system initialization, we considered two coordination shells: one of Ba and one of the SO_4_ tetrahedra. We used the cutoff distances: 4.1894 Å for the first coordination shells of Ba and SO_4_ (Ba-SO_4_ “bonds”), 6.6309 Å for the second coordination shell of Ba (Ba–Ba “bonds”), and 5.5927 Å for the second coordination shell of SO_4_ (SO_4_–SO_4_ “bonds”). The [001] crystal cleavage surface, which is the predominant surface in natural barite crystals, was used for the model. The supercell with periodic boundary conditions was generated by translating the unit cell along three crystallographic directions. The simulated system size corresponded to 200 × 400 × 30 unit cells, containing 5.76 × 10^5^ atoms.

**Figure 1:**
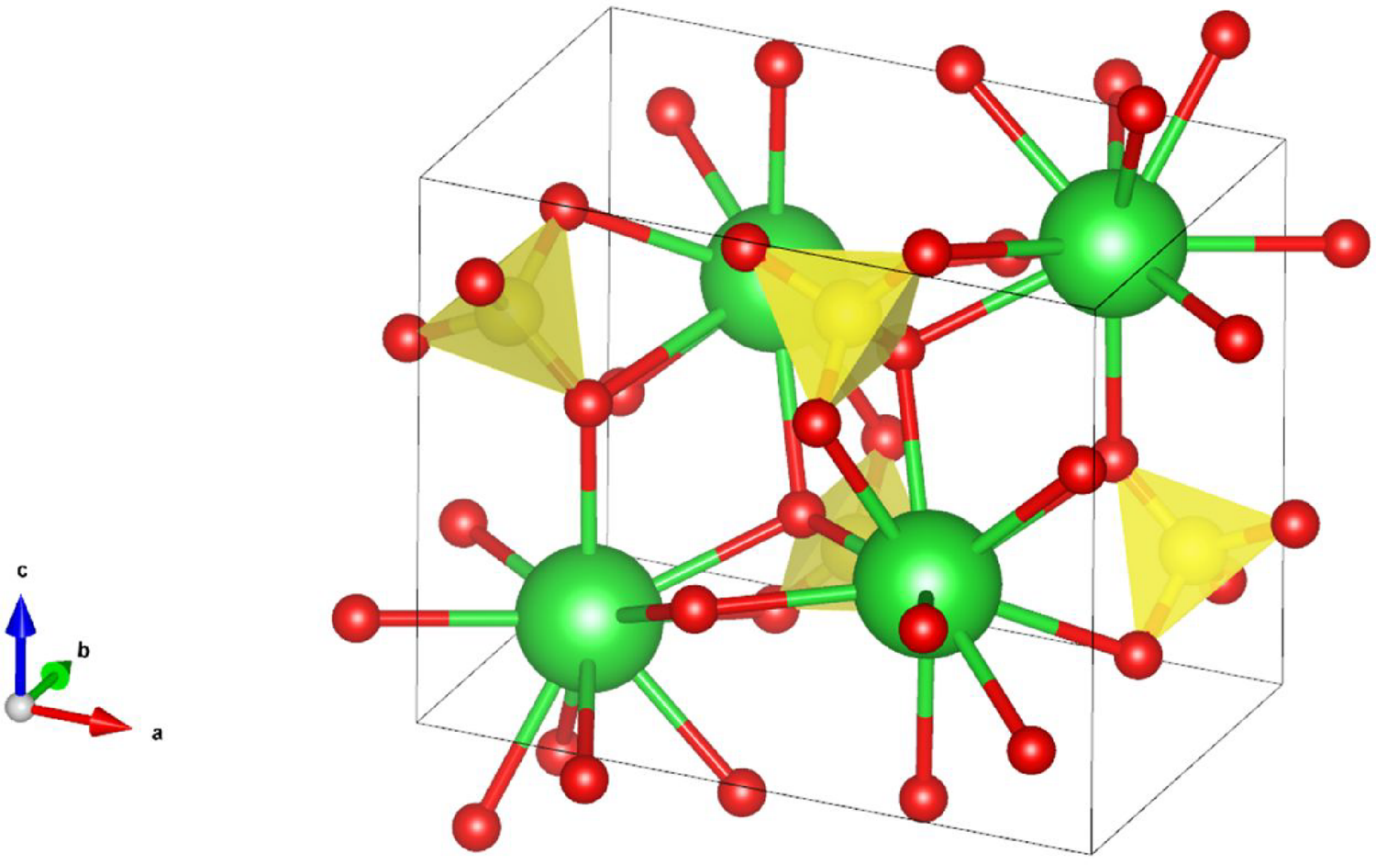
Unit cell of barite, ball-and-stick model with polyhedra. The barium atoms are colored green, oxygen red, and SO_4_ tetrahedra in yellow.

We used VMD software^90^ to visualize the.XYZ files of the reacted surfaces, and Vesta^91^ and Avogadro software (Version 1.2.0. http://avogadro.cc/)^92^ for visualizing the crystal structure.

### Kinetic Monte Carlo Model

#### Generic Algorithm

The idea of modeling a reactive system by stochastic methods was originally introduced by a seminal work of Gillespie.^68^ The kinetic Monte Carlo method allows us to model the temporal evolution of the system by random sampling over a set of all possible reaction events. The probability that a reaction event will be selected is directly proportional to its reaction rate.^71^ A decision regarding the selection of a certain event is made using a random number generator. The probabilities are calculated in a way that the selection procedure reproduces, on average, elementary reaction rates. In this study, one elementary reaction event corresponds to the dissolution of one surface atom. The individual system evolution trajectories, however, are stochastic.

In the kMC algorithm, the probability of one reaction event is directly proportional to its rate. The reaction probabilities are calculated by the normalization of certain reaction rates to the total sum of the reaction rates (eq 1)^68,93^

$$p_{i}=\frac{k_{i}}{\sum_{i=1}k_{i}}=\frac{k_{i}}{k\text{\_}\mathrm{tot}}$$ 1

where *p*_*i*_ *–* is the reaction event probability, *k*_*i*_ is the rate of one reaction event, and *k*_tot is the sum of all reaction event rates.

The reaction event selection procedure can be formulated in different ways. In this study, we used the standard BKL algorithm proposed for modeling the Ising-spin system.^93^ For choosing the reaction events, we used the so-called “divide-and-conquer”^93^ algorithm. The algorithm is implemented with the running sum method.^93^ The running sum is constructed in such a way that the probability of one reaction falls within the [0,1] range (Figure 2). Afterward, a uniformly distributed random number is used to select one reaction. The random number points to one reaction field, and this reaction occurs.

**Figure 2:**
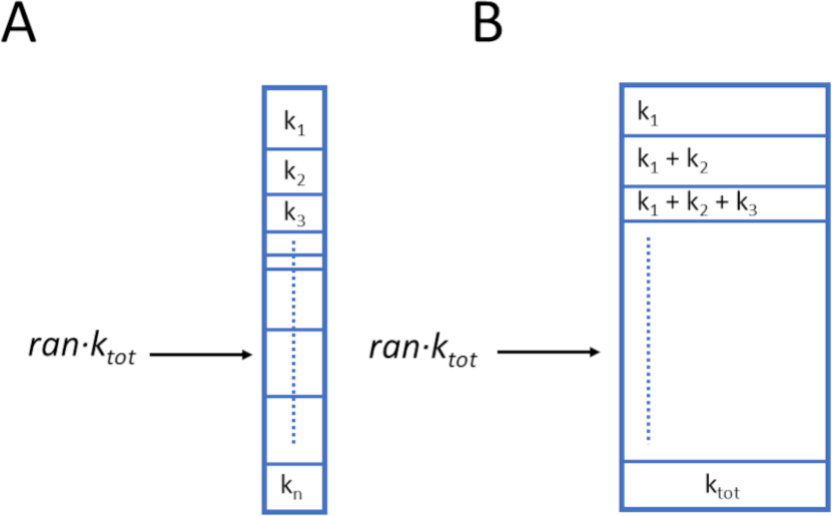
Principles of the running sum algorithm. (A) The box size corresponds to the values of reaction rates. The larger a box is, the higher the rate. (B) Running sum algorithm. Each region in the picture corresponds to one reaction. For more details, see the work of Voter.^71^

For the time change over the simulation step, the following formula was used^93^

$$\Delta t=-\frac{1}{k_{\mathrm{tot}}}\times \ln\left(\mathrm{ran}\right)$$ 2

where Δ*t* is a time step, *k*_*tot*_ is the sum of the reaction rates of all reaction events presented in the system at the time step Δ*t*, and ln­(ran) is the natural logarithm of a uniformly distributed random number.

#### kMC Model of Barite Dissolution

##### Program Workflow

The schematic flowchart (Chart 1) illustrates the organization of our code. The program reads input files containing information about the atomic coordinates and the configurations of neighboring atoms within a unit cell. Then, the program generates a supercell with lateral periodic boundary conditions and initializes the [001] cleavage surface plane. A user can introduce one or more point defects or hollow cores at this step. The diameter of a hollow core in the current simulations is set to one ion. Ba and one underlying SO_4_ tetrahedron are removed in the upper and lower parts of the unit cell, respectively, for the whole size of the system in the *c* direction. Afterward, the program launches the iterative loop for the user-specified number of iterations. At each iteration, the program (re)­calculates both the reaction rates and probabilities. After an atom has been removed, the program updates the local neighborhood. After performing all prescribed iterations, the program exports the output, which includes files containing surface site populations, material flux, step velocities, and surface morphology as well as an.XYZ file of surface atoms.

**Chart 1:**
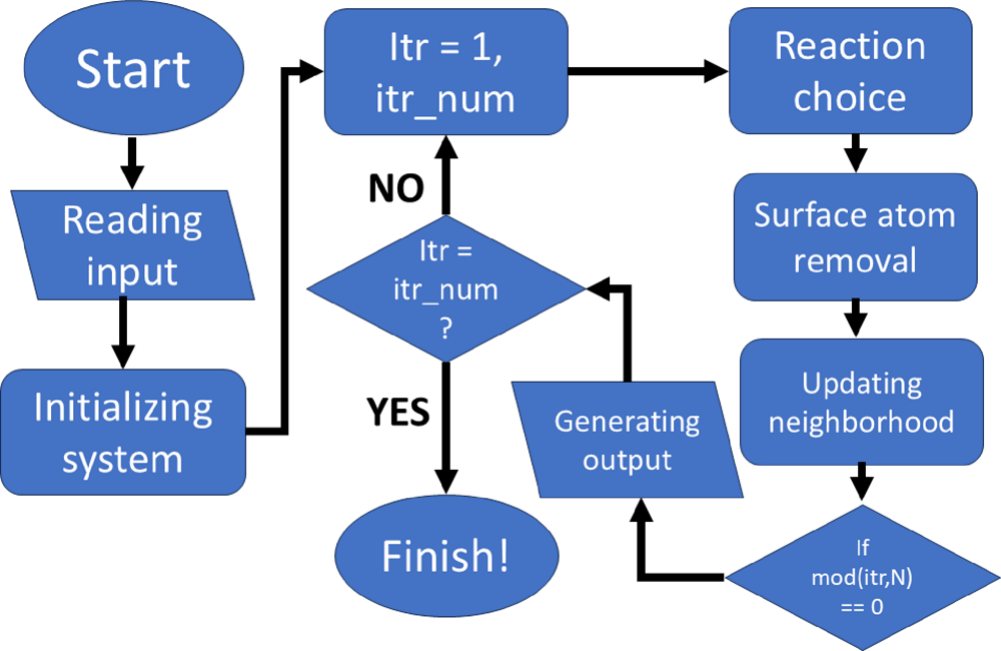
Workflow Chart for Our Kinetic Monte Carlo Program^a^

a Here, Itr is the iteration, itr_num is the number of iterations, *N* is the user-specified number of iterations after which the output files get generated, and mod is the module of the number.

##### Model Parametrization

One of the essential steps in developing the kMC model is constructing the correct ensemble of molecular reactions. The rates of these reactions are the input parameters. Therefore, their values should be obtained externally, e.g., by using *ab initio* or molecular dynamics calculations. However, it is often difficult to obtain the entirely determined parameter set due to the large number of possible reactions in a complex ensemble. In this case, the approach to estimate the values can come intuitively or heuristically by validating simulation results with the help of experimental results cp.^86,87^ Parameters such as mono- and multilayer etch pits’ morphology and kinetics of etch pit steps can be utilized to parametrize the model against experimental data.

The natural shape of the monolayer barite etch pit, which was taken as a reference for the parametrization procedure, is shown in Figure 3A.^94^ We added parameters related to molecular reaction rates and structural controls stepwise and tested the output results against experimental data. The final and intermediate parameter sets for reaction rates calculation are listed in Table 1. At the first step of the parametrization procedure, we added only the first coordination shell neighbors for Ba and SO_4_ tetrahedra, which resulted in the formation of an oval-shaped etch pit (Figure 3B).

**Figure 3:**
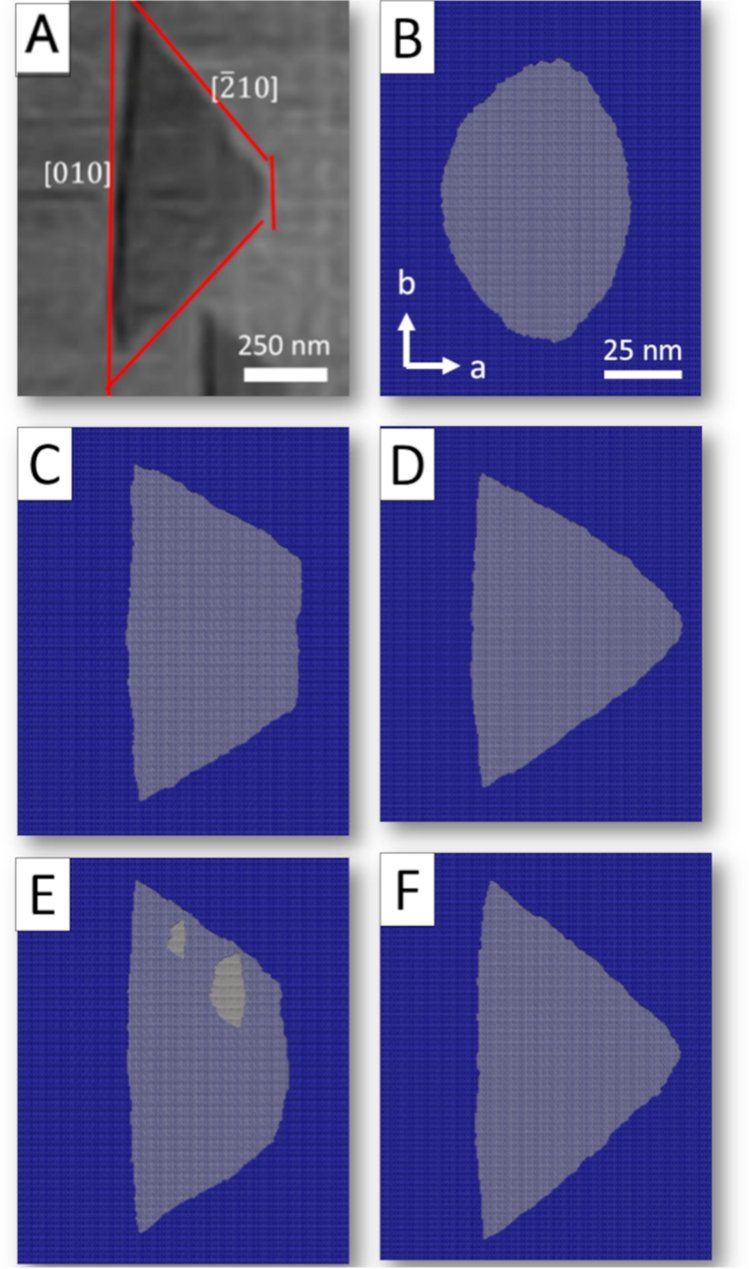
Results of simulations of the monolayer etch pit morphology obtained by using different parameter sets. (A) Monolayer etch pit morphology was obtained from experimental AFM studies (Kuwahara, 2012) (Am. Min., 97, 1564–1573, Copyright (2012), MSA, reproduced with the permission of the Mineralogical Society of America). (B–F) Etch pit morphologies at the parameter sets B, C, D, E, and F, respectively. The orientation of the surface is the same for all of the pictures. The system’s size is equal for all the figure panels except panel (A).

**Table 1:** Parameter Set Used for Step-by-Step Parameterization^a^

parameter	set B	set C	set D	set E	set F
*V*	10^10^	10^10^	10^10^	10^10^	10^10^
Δ*E* Ba–O–S	15.5	15.5	15.5	15.5	15.5
Δ*E* Ba–O–Ba	0	1.2	1.2	1.2	1.2
Δ*E* S–O–Ba	15.7	15.7	15.7	15.7	15.7
*kw* Ba–O–S	0	0	0	3.7	3.7
*kw* Ba–O–Ba	0	0	0	5.3	5.3
*kw* S–O–Ba	0	0	0	3.7	3.7
*Ws* Ba_4–9_	0	0	6.5	0	6.5
*Ws* Ba_3–7_	0	0	4.0	0	4.0
*Ws* S_4–7_	0	0	4.0	0	4.0
*Ws* S_3–7_	0	0	6.0	0	6.0
*Wt*	–200	–200	–200	–200	–200

a All values except *v* are given in kJ/mol units; the *v* parameter is given in Hz.

According to our previous study,^88^ the second coordination shell of Ba has a significant influence on the activation barrier for Ba ion detachment. After the addition of the second coordination shell of Ba to the parametrization procedure, we obtained a trapezoidal etch pit with the stable [010]-fast step (Figure 3C). However, in a natural etch pit, the fast [010] step is unstable. Therefore, we assumed that the reactivity of the step sites at the [010]-fast step is increased by site-specific steric factors. We considered the stable neighbors’ configurations of the [010]-fast step and increased their reaction rates. These configurations correspond to Ba 4–9 and Ba 3–7 ions, SO_4_ 4–7 and SO_4_ 3–7 ions, where the first value is the number of neighbors in the first coordination shell and the second number of neighbors in the second coordination shell (Ws_Ba_4–9_, Ws_Ba_3–7_, Ws_S_4–7_, Ws_S_3–7_ in Table 1). These coefficients, which increase the reactivity of surface sites, were applied to the entire system and not just the [010]-fast step. After introducing steric factors into the model, we obtained a triangular etch pit that was slightly rounded compared to the natural one (Figure 3D).

At the next step of parametrization, we tested the hypothesis that the activation energies should be normalized by the bond lengths to implicitly introduce the effect of Coulomb interactions (eq 3). First, we applied the parametrization using bond lengths without introducing steric factors. We introduced weighting coefficients (kw coefficients in the Table) to normalize the activation energies by bond length. These coefficients allowed us to vary the effect of short- and long-range order neighbors. By varying these coefficients, we observed that the most reliable coefficient values correspond to the mean value among all bond lengths within the coordination shell. After introducing the bond lengths, we obtained a trapezoidal etch pit similar to the one that we obtained without adding the bond lengths to the parametrization procedure (Figure 3E). The resulting etch pit, however, had a slightly less stable [010]-fast step. At the next parametrization step, we applied the same steric factors and obtained the completely reproduced natural shape of an etch pit (Figure 3F).

$$\varphi \left(l\right)=k\frac{q}{l}k=\frac{1}{4\pi \varepsilon_{0}}$$ 3

After reproducing the triangular shape of an etch pit, we calculated the step velocities at various temperatures and compared them with experimentally obtained data.^94,95^ At the elevated temperatures, our data showed high disagreement with the data of Kuwahara,^94^ who measured the monolayer step velocities at various temperatures. We proposed that the water attempt frequency *v* = 10^12^, proposed by Pelmenschikov et al.^96^ for silicates, is too high for ionic solids. We tested the values of *v* = 10^11^ and *v* = 10^10^ and concluded that by choosing *v* = 10^10^, the experimental results and our results fit within the margin of error. After the *v* parameter was set to 10^10^, our data matched the experimental data almost within the margin of error.

##### Calculation of the Reaction Rates

The reaction rates for barium atoms were calculated considering first coordination shell containing 7 SO_4_ tetrahedra, and the second coordination shell containing 14 Ba atoms:

$$k_{i,j}^{A}=v\times Wt\times W_{s}\times \exp\left(-\left(i\frac{\Delta E_{\mathrm{Ba}-{\mathrm{SO}}_{4}}kw_{\mathrm{Ba}-{\mathrm{SO}}_{4}}}{kTl_{\mathrm{Ba}-{\mathrm{SO}}_{4}}}+j\frac{\Delta E_{\mathrm{Ba}-\mathrm{Ba}}kw_{\mathrm{Ba}-\mathrm{Ba}}}{kTl_{\mathrm{Ba}-\mathrm{Ba}}}\right)\right)$$ 4

For the SO_4_ groups, we assumed that only the influence of the seven Ba atoms of the first coordination shell significantly affects the dissolution process. To distinguish between kink and step sites, we added the second coordination shell of SO_4_ tetrahedra, containing 12 SO_4_ groups, to the model. However, it had no effect on the reaction rates.

$$k_{i}^{{\mathrm{SO}}_{4}}=v\times Wt\times W_{s}\times \exp\left(-i\frac{\Delta E_{{\mathrm{SO}}_{4}-\mathrm{Ba}}kw_{{\mathrm{SO}}_{4}-\mathrm{Ba}}}{kTl_{{\mathrm{SO}}_{4}-\mathrm{Ba}}}\right)$$ 5

where *v* = 10^10^ Hz– is the approximated water attempt frequency, *Wt* – is a factor, which reduces the terrace sites reactivity, Δ*E* is an activation energy factor of bond breaking, *k* is the Boltzmann constant, *T* is the temperature in degrees Kelvin, and *l* is the interatomic distance between reacting atom and its neighbors (Ba-SO_4_, SO_4_–Ba, and Ba–Ba).

The *Wt* factor was introduced to model far-from-equilibrium conditions and avoid the formation of multiple etch pits on the surface:

$$Wt=\exp\left(\frac{-w}{kT}\right)$$ 6

The *Ws* parameters were used to introduce steric factors, which increase reactivity for some surface sites due to their specific water coordination.

$$W_{s}=\exp\left(\frac{w}{kT}\right)$$ 7

By introducing the effect of the bond lengths on the dissolution rates, we used the normalization coefficients *kw*_Ba–SO_4__, *kw*_Ba–Ba_, and *kw*_SO_4_–Ba_, which allow us to vary the effect of the short- and long-range order atoms within one coordination shell. The parameters we used for the model parametrization are listed in Table 1 below.

The model does not consider atomic attachment and surface diffusion, focusing solely on the dissolution process under far-from-equilibrium conditions. Although these two processes are very important for realistic scenarios of pore spaces in geological reservoirs, their addition may change the process mechanisms. The goal of this study is to investigate first the basic structure-driven mechanisms of barite dissolution. Adding these processes and investigations of their effects should be a subject of separate studies and publications.

More extensive explanations of how kMC models are developed, parametrized, and verified are provided in our previous publications and references therein.^84–88^

### Data Analysis

#### Step Velocities

The step velocities were calculated by the formula:

$$v_{\mathrm{step}}=\frac{\Delta l}{\Delta t}$$ 8

where *v*_step_ is a monatomic step velocity, Δ*l* is the linear step propagation difference, and Δ*t* is the time difference.

The Arrhenius equation describes the reaction rate change with temperature:

$$r=v\times \exp\left(-\frac{A}{kT}\right)$$ 9

where *r* is the reaction rate, *v* is the preexponential factor, *A* is the activation energy, *k* is the Boltzmann constant, and *T* is the temperature in degrees Kelvin.

Kinetic parameters, such as step velocity or material flux, also change exponentially with temperature due to their relationship to the molecular reaction rates.

#### Material Flux

We calculated the material flux over time trajectories *M*(*t*_*j*_) by calculating the cumulative numbers of dissolved atoms at every 10,000 iteration steps and the time elapsed between two consecutive tabulated values. Each tabulated value is designated as the *j* index. We divided the difference between those cumulative numbers by the time step elapsed and normalized the result by the total surface area over a given time step and the Avogadro number to obtain mol/(cm^2^·s) units:

$$M\left(t_{j}\right)=\frac{m_{j+1}-m_{j}}{\left(t_{j+1}-t_{j}\right)}$$ 10

where *m*_*j*_ is the normalized number of dissolved atoms at time *t*_*j*_.

$$m=N/\left(N_{A}\cdot S\right)$$ 11

where *m* is the material flux, *N* is the number of dissolved atoms, *N*_*A*_ is Avogadro’s number, and *S* is the surface area in cm^2^. The surface area calculated is the initial surface area of the system.

$$S=na\times mb\times 10^{-16}$$ 12

where *n* and *m* are the number of unit cells in the supercell in the *a* and *b* directions, respectively, *a* and *b* are the unit cell parameters, and 10^–16^ is the number converting Å^2^ to cm^2^.

#### Statistical Treatment of the Data

##### Step Velocity

To calculate the monolayer step velocity values at different temperatures and their standard deviations at fixed temperatures, we run 10 simulations for identical conditions. We calculated the average step velocity (eq 13) and the standard deviations (eq 14) between ten different trajectories and presented their values in Table 2. We used the following formulas:

$$v\left(t_{j}\right)=\frac{\sum_{i=1}^{N}\frac{\Delta l_{j}}{\Delta t_{j}}}{N}$$ 13

where *v* is the step velocity, *N* is the number of trajectories (in our case, *N* = 10), Δ*l*_*j*_ is the total distance between the position of a propagated step at time *t*_*j*_ and the point defect, placed in the center of the system, and Δ*t*_j_ is the time it takes for the step to cover the distance Δ*l*_*j*_.

$$s=\sqrt{\frac{\sum {\left(v_{i}-v_{av}\right)}^{2}}{N}}$$ 14

where *s* is a standard deviation, *v*_*i*_ is a step velocity in one trajectory, *v*_*av*_ is the average step velocity among ten trajectories, and *N* is the number of trajectories (in our case, *N* = 10).

**Table 2:** Step Velocities for Steps of Triangular Etch Pit *n ×* 10^–1^ nm·s^–1^ ^a^

reference	*T* (°C)	[010] slow	[010] fast	[010] total	[120]	[2̅10]
Risthaus (2001)	22			0.3 ± 0.1		
this study	22	0.052 ± 0.002	0.101 ± 0.001	0.153 ± 0.01	0.077 ± 0.002	0.077 ± 0.003
Kuwahara 2011	30	0.095 ± 0.005			0.15 ± 0.01	0.15 ± 0.01
Kuwahara 2012	30	0.10 ± 0.01			0.15 ± 0.01	0.15 ± 0.01
this study	30	0.10 ± 0.01	0.20 ± 0.051	0.31 ± 0.01	0.16 ± 0.01	0.16 ± 0.01
Kuwahara 2012	40	0.20 ± 0.05			0.31 ± 0.05	0.31 ± 0.05
this study	40	0.25 ± 0.01	0.47 ± 0.01	0.73 ± 0.01	0.37 ± 0.01	0.37 ± 0.01
Kuwahara 2012	55	0.7 ± 0.1			1.0 ± 0.1	1.0 ± 0.1
this study	55	0.85 ± 0.02	1.54 ± 0.03	2.39 ± 0.04	1.22 ± 0.04	1.22 ± 0.04
this study	90	9.7 ± 0.3	15.9 ± 0.3	25.6 ± 0.4	13.2 ± 0.4	13.0 ± 0.3

a The accuracy of our data corresponds to the accuracy of the experimentally obtained data. In our kMC data at higher temperatures, the error intervals decrease due to an increase in the natural fluctuations of the system.

This procedure to treat the step velocities was chosen due to its similarity to the direct step velocity measurement, provided by the AFM experiments.^25,94,95^ It also ensures robustness of values, which is important for our small systems where large stochastic fluctuations of time steps make time-dependent analysis problematic for small Δ*l*_*j*_. The data presented in Table 2 represent the last values of the step velocities where they reached the steady state and where the change of the step velocity value was less than the standard deviation (approximately 3–5% of the step velocity value). An example of the temporal dependence of cumulative step velocities on time and an example of standard deviation behavior over time are shown in the Supporting Information, Figures S1 and S2.

##### Material Flux

The average trajectories of the material fluxes between ten runs at identical conditions were calculated according to eq 15.

$$M_{av}\left(t_{j}\right)=\frac{(\sum_{i=1}^{N}M_{i}\left(t_{j}\right)}{N}$$ 15

where *M*_*av*_ is an average material flux trajectory, *M*_*i*_ is a single trajectory, and *N* is the number of trajectories (in our case, *N* = 10).

The standard deviations of the material fluxes between ten trajectories were calculated at different time steps (eq 16).

$$s\left(t_{j}\right)=\sqrt{\frac{\sum_{i=1}^{N}{\left(M_{i}\left(t_{j}\right)-M_{av}\left(t_{j}\right)\right)}^{2}}{N}}$$ 16

where *s*(*t*_*j*_) is the standard deviation, *M*_*i*_ is one trajectory of the material flux, *M*_*av*_ is the average of the material flux over all trajectories, and N is the number of trajectories (10 for our case).

The data in the subsection ″Change in Material Flux with Temperature” are represented as the average of the entire averaged material flux trajectory over 10 simulation runs, with the maximal value of the standard deviation obtained over these 10 simulation runs.

The material flux trajectories along with the plotted ranges of standard deviations are provided in Supporting Information, Figures S3–S9.

## Results and Discussion

### Mechanisms

#### Monolayer Etch Pits

The monolayer etch pit has a triangular shape controlled by [120], [2̅10], and [010] crystallographic directions. Four steps define the shape of the etch pit: the [010]-slow, [010]-fast, [120], and [2̅10] steps (Figure 4A). Due to the presence of 2_1_ axes in the barite crystal structure, the multilayer etch pit′s shape is determined by the superposition of triangular pits, which rotate by 180° (Figure 4B).

**Figure 4:**
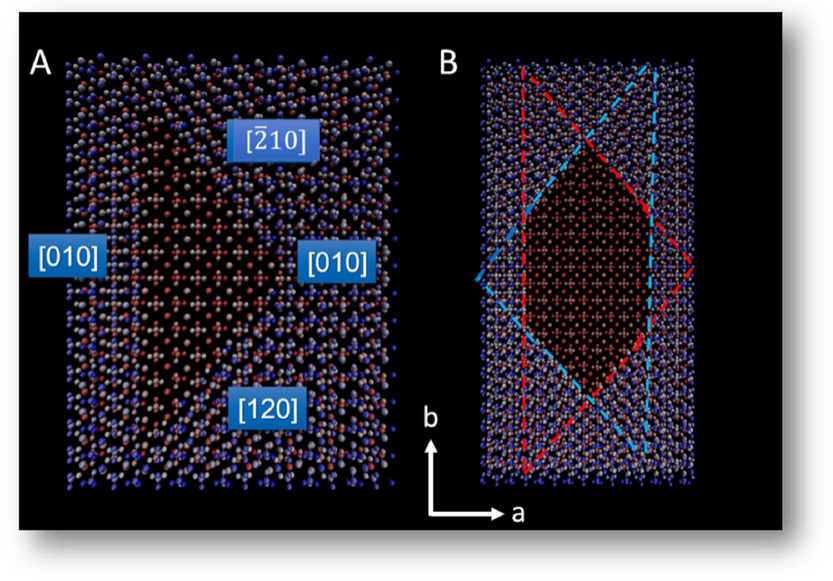
Morphology of monolayer (A) and bilayer (B) etch pits. Due to the presence of the 2_1_ screw axis in the structure of barite, the shape of a bilayer etch pit is a result of the superposition of triangular monolayer etch pits.

We calculated the step velocities for [010] (slow), [010] (fast), [120], and [2̅10] steps of the monolayer etch pit obtained in our model and compared their values with the experimental data (Table 2).^25,94,95^

The step velocities obtained in the kMC simulation reproduce the experimental results at 30 °C and closely match the experimental measurement errors at 40 and 55 °C. We extrapolated the temperature range up to 90 °C. The extrapolation above 100 °C is possible. However, we would not interpret this extrapolation as reliable due to the changes in water properties above the boiling point. The water behavior changes at elevated temperatures, e.g., the electric properties of water and hydrogen bonding, especially above the boiling point. We assume that these changes do not significantly affect the model’s predictive power. We suggest that the system’s behavior below the boiling point of water can be sufficiently described by the Arrhenius equation (eq 9).

The step velocities of [120] and [2̅10] showed a slight difference at 90 °C, resulting in an asymmetry of the etch pit’s shape (Table 2). The difference in step velocities means that the kink sites at one step propagate faster than those at another step, resulting in one step being longer than the other. The [120] and [2̅10] directions in the barite lattice structure are completely equivalent in terms of generating the same periodic bond chains. However, an asymmetry of atomic steps with the domination of one of them can occur in natural etch pits, as shown in Figure 5. We suspect that the difference in the step velocities can be caused by the stochastic component. In our data, the difference in the step velocities stays within the margin of error. We suggest that the asymmetry of the etch pits obtained in the experiments can be caused by stochastic fluctuations in the detachment of the kink sites from the atomic steps.

**Figure 5:**
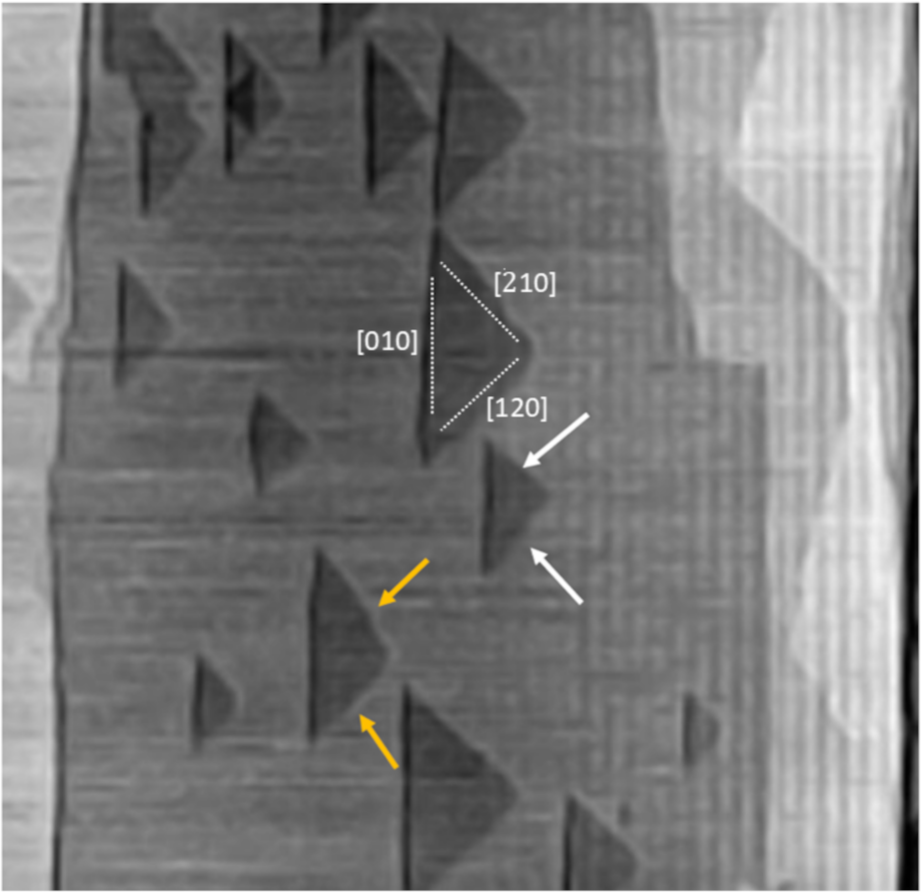
Monolayer etch pits on a barite surface in water at 55 °C. The white and yellow arrows point to the steps of an etch pit with faster [120] and [2 ®10] step retreat rates, respectively. The white dotted lines show the crystallographic directions of the steps. The crystallographic indexes are given in white [Reprinted (adapted or reprinted in part) with permission from Kuwahara, Y. (2012). In situ hot-stage AFM study of the dissolution of the barite (001) surface in water at 30–55 C. *American Mineralogist*, 97(10), 1564–1573. Copyright 2012 Mineralogical Society of America).

#### Multilayer Etch Pits

The shape of a multilayer etch pit is determined by the superposition of monolayer etch pits, rotated by 180° within two monatomic half-unit-cell layers. The multilayer etch pit is formed by steps along the [010], [120], and [−210] directions. The multilayer etch pits’ morphology reported in the experimental studies^25,94,95,97^ was completely reproduced in the kMC model.

The [010] step is formed by [010] slow and [010]-fast steps. The step propagation is limited by the retreat of the [010]-slow step, with subsequent propagation of the [010]-fast step. The [010]-fast step encounters the [010]-slow step, resulting in the formation of doubled [010] steps (Figure 6A,C, zone A). The [120] and [2̅10] steps are crystallographically equivalent. Their propagation is controlled by the propagation of a [010]-fast step. This is the reason why they also form the doubled steps (Figure 6A,C, zone B). However, at the intersection between the single [120] and [2̅10] steps, the double step splits into two independent steps and forms zigzag patterns (Figure 6A,C, zone C). The zigzag patterns at the layers’ intersection are characteristic of minerals that have a rotation axis within the unit cell and have been reported in experimental^98,99^ and modeling^86^ studies.

**Figure 6:**
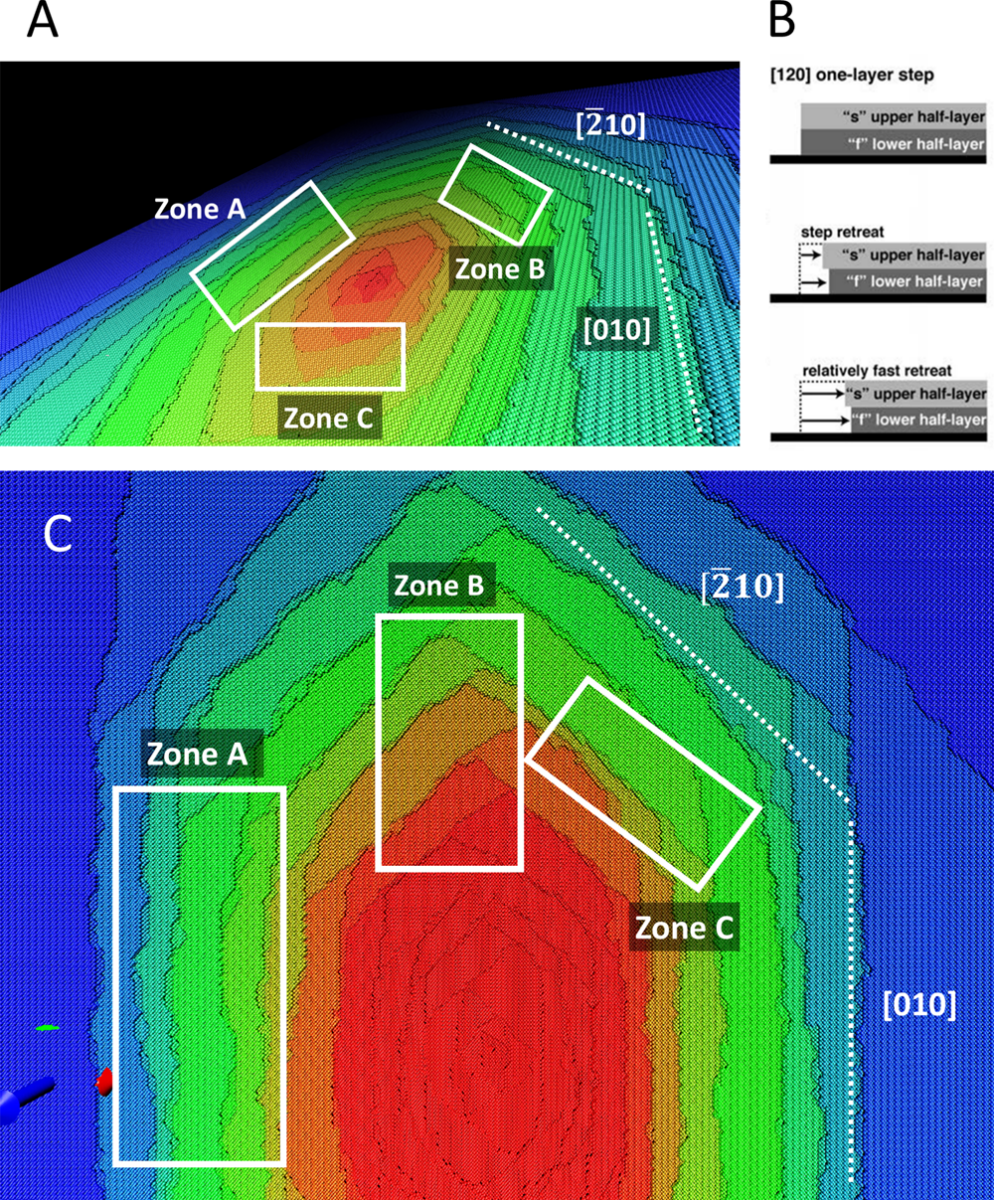
Mechanisms of step propagation in the barite multilayer etch pit. (A) Zoomed image of the multilayer etch pit produced by the kMC simulation at 22 °C. B Model of the [120] step retreat proposed by Kuwahara^94^ (Reprinted (adapted or reprinted in part) with permission from Kuwahara, Y. (2012). In situ hot-stage AFM study of the dissolution of the barite (001) surface in water at 30–55 C. American Mineralogist, 97(10), 1564–1573. Copyright 2012 Mineralogical Society of America). (C) Zoomed image of the multilayer etch pit (orthogonal view).

In his study, Kuwahara^94^ proposed a mechanism of [120] and [2̅10] steps retreat via the initial propagation of the fast step in the lower layer, promoting the subsequent propagation of the slow step in the upper layer, with the formation of a “bench” (Figure 6B). We did not observe this behavior of the steps in our study and conclude that the detachment of atoms from the lower step is possible but not preferred. The atomic detachment from the lower layer, with the formation of a “bench”, requires higher energy costs due to the high number of bonds that must be broken. The atoms at the upper step are less coordinated than those at the lower ones. Ba atoms at the upper step have three fewer neighbors in the first coordination shell and seven fewer neighbors in the second coordination shell compared to Ba atoms at the lower step. Sulfate tetrahedra follow the same trend, having one fewer neighbor in the first coordination shell and three fewer neighbors in the second coordination shell at the upper step compared to the SO_4_ tetrahedra at the lower step. The retreat of the upper slow layer, followed by fast removal of the lower layer, appears to be energetically more favorable.

#### Changes in the Etch Pit Morphology with Temperature

The temperature affects the shape of the etch pits. The shape of the monolayer etch pits becomes more rounded as the temperature changes from 30 to 95 °C (Figure 7A). The same trend is observed for the multilayer etch pits (Figure 7B). Their shape changes to be more rounded and rougher in comparison to the etch pits formed under ambient conditions. Additionally, with the increase in temperature, they become deeper and smaller in the lateral directions (Figure 7B).

**Figure 7:**
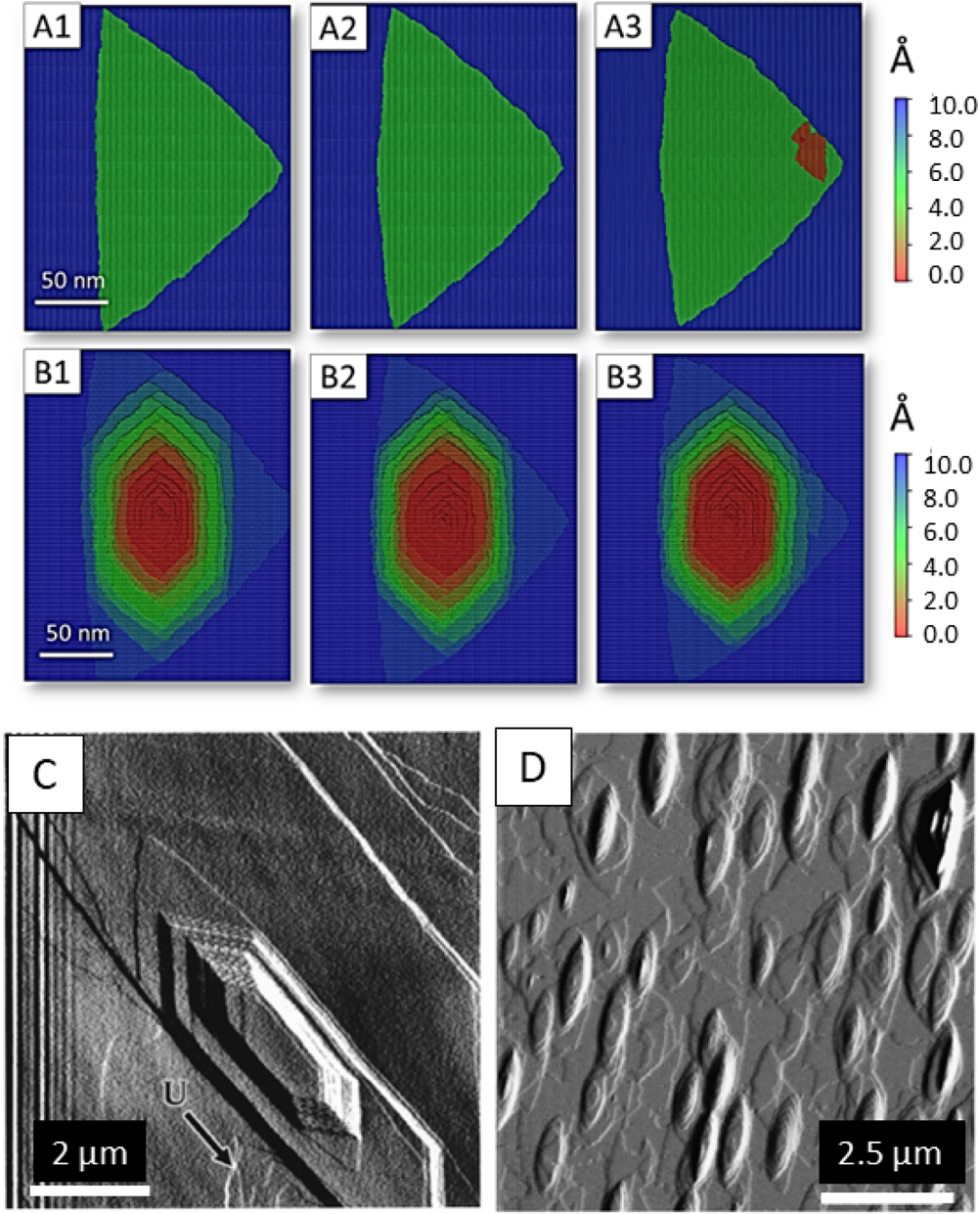
Change of etch pit morphology with temperature. (A, B) kMC simulations. (A1, A2, A3) Monolayer pit at 30, 55, and 90 °C, respectively. (B1, B2, B3) Multilayer etch pit at 30, 55, and 90 °C, respectively. (C, D) AFM experiments. The shape of the multilayer etch pit at (C) 125 °C. Reprinted (adapted or reprinted in part) with permission from Higgins, S. R., Jordan, G., Eggleston, C. M., & Knauss, K. G. (1998). Dissolution kinetics of the barium sulfate (001) surface by hydrothermal atomic force microscopy. *Langmuir*, 14(18), 4967–4971. Copyright 1998 American Chemical Society. (D) 60 °C. Reprinted (adapted or reprinted in part) with permission from Kowacz, M., & Putnis, A. (2008). The effect of specific background electrolytes on water structure and solute hydration: Consequences for crystal dissolution and growth. *Geochimica et Cosmochimica Acta*, 72(18), 4476–4487. Copyright 2008, Elsevier.

These observations are in agreement with Kowacz and Putnis,^24^ who obtained AFM images of rounded etch pits at 60 °C (Figure 7D). However, our results are not in agreement with the observations made by Higgins et al.^97^ According to their work, the etch pits are more elongated and have straight steps at 125 °C (Figure 7C). We suspect that this can be explained by the change in water properties above the boiling point and the increase in steric hindrance. In Figure 7C, created by Higgins, the etch pit is formed near the cleavage step and the [010] (slow) step of a triangular pit. It restricts the growth of an etch pit, resulting in subsequent elongation along the [010] direction. Overall, we conclude that the changing of etch pits’ shape with temperature is caused by reducing the difference in kink site detachment rates and altering the ratio of kink site generation to kink site propagation. It indicates the predominant role of the kink sites in the overall dissolution process.

#### Interaction of Multilayer Etch Pits

The idea that the etch pits open at the centers of dislocations was originally developed by Lasaga and co-workers.^69,70,100^ The etch pits are the primary source of atomic steps at locations far from grain boundaries. Lasaga and Luttge^70,101^ presented the so-called stepwave model of crystal dissolution. This model considered the multilayer etch pits to be the primary source of the steps, whose behavior exhibited similarities to the propagation of waves and their interactions. The centers of the stepwaves are the multilayer etch pits, presented in the centers of the hollow cores. The interaction of the etch pits can provide valuable insights into the mechanisms of mineral dissolution.

We studied the interaction mechanisms of multilayer etch pits on a barite surface by running long-trajectory simulations for systems with multiple hollow cores. In each simulation run, we initialized the system with ten randomly spatially distributed hollow cores. Figure 9 shows that only a few of the ten hollow cores are active and produce the stepwaves. The interaction of the multiple etch pits generated by hollow cores was studied by Kurganskaya and Luttge.^102^ They concluded that a broad range of different dissolution regimes can occur, independent of the number of hollow cores. Figure 8 shows an AFM image of dissolving barite. The etch pits are distributed linearly in the [010] and [120] directions. The etch pits from multiple sources merge and form a common dissolution front. The similar behavior of the etch pits obtained in our simulation is illustrated in Figure 9B. In Figure 9A, the interaction between the two dominant etch pits is demonstrated. As reported by Kurganskaya and Luttge (2023), in this situation, one etch pit can become dominant, while another can become dominant in its place. The one dominating etch pit is shown in Figure 9C. This etch pit interacts with itself due to periodic boundary conditions. This situation can occur for any number and relative positions of the hollow cores.^102^ Figure 9D illustrates the scenario where multiple hollow cores form a merged etch pit with several step sources. In this situation, a single etch pit of a complex morphology is formed. The morphology of the resulting etch pit depends on the number of active hollow cores, their mutual position, and the dominance regime of the etch pits, which is determined by a stochastic component.

**Figure 8:**
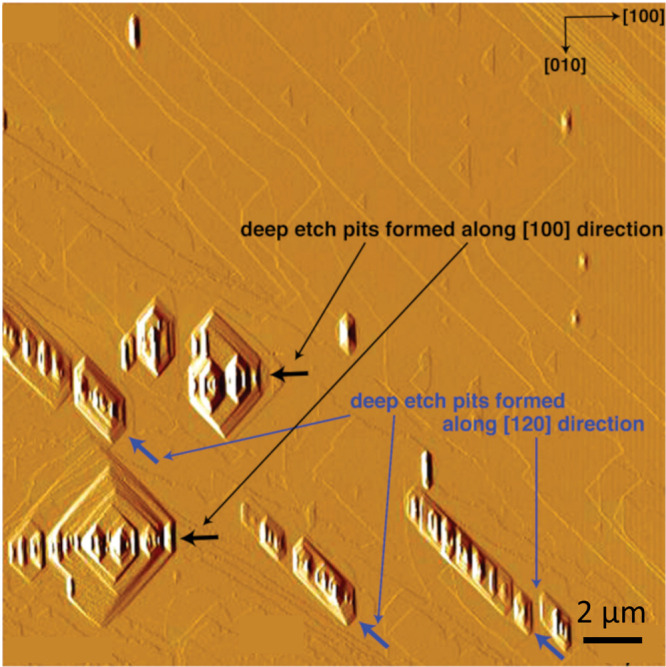
Interactions of the multilayer etch pits forming around several sources^94^ (Reprinted (adapted or reprinted in part) with permission from Kuwahara, Y. (2012). In situ hot-stage AFM study of the dissolution of the Barite (001) surface in water at 30–55 C. American Mineralogist, 97(10), 1564–1573. Copyright 2012 mineralogical Society of America).

**Figure 9:**
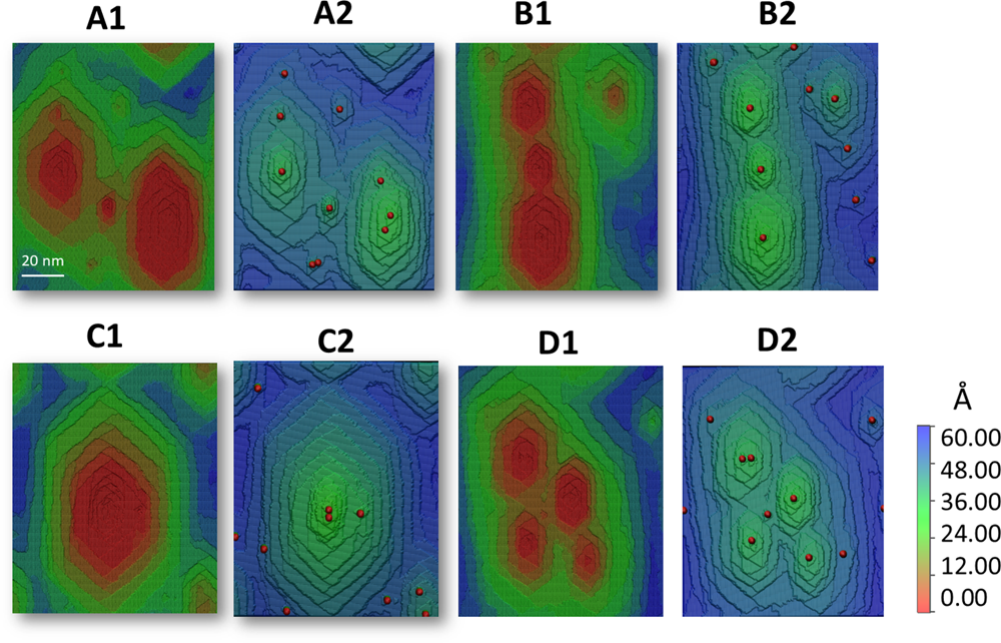
Morphology of the etch pits obtained by running simulations for four different systems with 10 randomly positioned hollow cores. (A–D) Different runs, resulting in different morphologies. (1) Surface morphology. (2) Surface map with marked positions of the hollow cores (red dots).

### Material Flux

#### Temporal Oscillatory Dynamics

Studies of material flux from the mineral surface are essential for predicting mineral dissolution under various conditions. We ran a series of simulations to determine the value of the material flux at various hollow core densities and temperatures. In cases where we ran long-time trajectories with the presence of more than one hollow core, we observed oscillations in the material flux over time (Figure 10). To better demonstrate this phenomenon, we discuss here the temporal structure of a material flux generated in the system with ten randomly distributed hollow cores.

**Figure 10:**
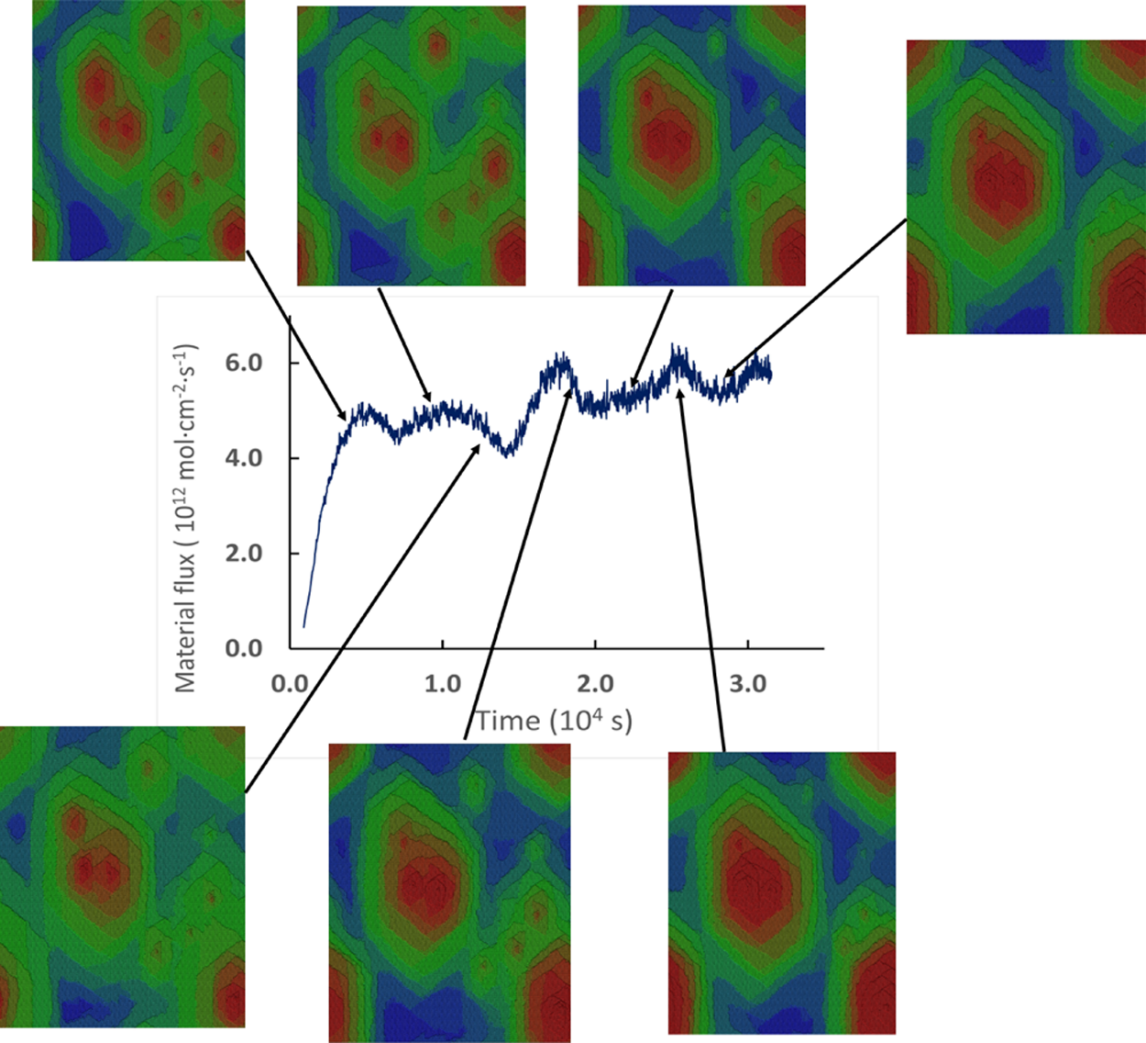
Material flux plot showing temporal oscillations in the system with 10 randomly distributed hollow cores. The pictures show snapshots of the system’s surface at different moments in time. The plot and snapshots are obtained from one kMC simulation.

The plots of material flux showed quasi-periodic oscillations, which differed for each simulation run. The oscillations of the material flux can be explained in terms of etch pit interactions. Similar behavior of material flux was found by using both experimental^103,104^ and computational techniques.^85,102,105^

The maximal values of material flux are reached when, due to the interaction of multilayer etch pits, curved fast steps are formed. Jordan and Rammensee^106^ observed the curved steps formed by the interacting etch pits on the calcite surface. They found that these steps significantly contribute to the overall dissolution rate, approximately by an order of magnitude, compared with the straight steps. The contribution of “curved” steps to the material flux is so high because their kink site densities are significantly higher than those of straight steps. These higher densities are covered by our kMC approach and are observed on the surface topography maps. The maximal effect of curved steps is observed when the etch pits merge at the same height level. After the curved steps induce the retreat of single or multiple layers, the material flux reduces to a minimum, and the process of generating curved steps starts again. Temporal oscillations of material fluxes have been observed in various systems. Kurganskaya and Luttge^85^ found oscillations in material flux for the calcite surface with interacting etch pits forming around hollow cores. The authors attributed the temporal fluctuations to fluctuations in kink site densities and also identified the specific kink sites, whose detachment drove the dissolution. Fisher and coauthors^105^ reported the oscillatory pattern of material flux occurring in a Kossel crystal. The maximum oscillations were reached when a surface was maximally rough and the curved steps dominated. When the surface becomes flatter, the oscillations reach the minima. Kurganskaya and Luttge^102^ studied the effect of the distribution of hollow cores on dissolution for the Kossel crystal. They found that by maintaining the same initial configuration of the hollow cores, the system can transition through a wide range of different regimes, e.g., with one, two, or several dominating etch pits, each having a distinct impact on the dissolution rate. The type of regime was controlled not only by the hollow core numbers and locations but also by the stochastic component, which determined which regime would occur at a distinct moment of time.

The quasi-periodic oscillations occur by barite dissolution and have similarities as well as differences in comparison to those of Kossel crystals or calcite. While in calcite and Kossel crystals the oscillations have an entirely stochastic behavior, in the case of barite, the system is particularly deterministic. The maxima and the minima on the material flux plot are determined not only by the presence or absence of curved steps, but also by the shifting of the system from one dissolution regime to another in a way similar to those described by Kurganskaya and Luttge^102^ for Kossel crystals. The oscillations observed for the barite (001) surface, however, are principally different from those observed for the Kossel and calcite crystals. First, the periods of oscillations are much longer, and the period length fluctuates much less. In some sense, they can thus be considered to be almost quasi-periodic. Second, long-period oscillations significantly dominate short-term oscillations, which are induced by the formation of curved steps. This difference may stem from the existence of 2_1_ rotation screw axis in the barite structure, which enables the domination of the slowest steps and processes.

#### Dependence of the Material Flux on the Dislocation Density

The effect of the dislocation density on mineral dissolution rates has been widely studied. The idea that the dislocations significantly affect the dissolution process was proposed by Holdren and Berner.^107^ In many studies, the strong effect of the dislocation density on the mineral dissolution rates is observed, e.g.,.^108–114^ Other authors reported the relatively small effect of dislocation on the dissolution process.^115–117^ Holdren and coauthors^118^ reported an increase in the dissolution rate of plagioclase three times, corresponding to a dislocation density increase from 10^6^ to 10^9^ cm^–2^. Murphy^119,120^ observed no change in dissolution rate with variations in dislocation densities (from 10^6^ to 10^9^ cm^–2^) in sanidine.

To study the effect of dislocation density on the total material flux from the surface, we ran a series of ten simulations for each dislocation density and calculated the average material flux. The modeled dislocation densities 1 × 10^7^, 3 × 10^7^, 5 × 10^7^, and 10 × 10^7^ dislocations per μm^2^ correspond to one, three, five, and ten hollow cores in the system, respectively. One limitation of our study in comparison to the literature is that we consider significantly higher dislocation densities per unit area. The plots of material flux over time for all four dislocation densities are presented in Figure 11. The values of material flux oscillate within the range of 3.5·10^–12^ to 4.0·10^–12^ mol·cm^–2^·s^–1^.

**Figure 11:**
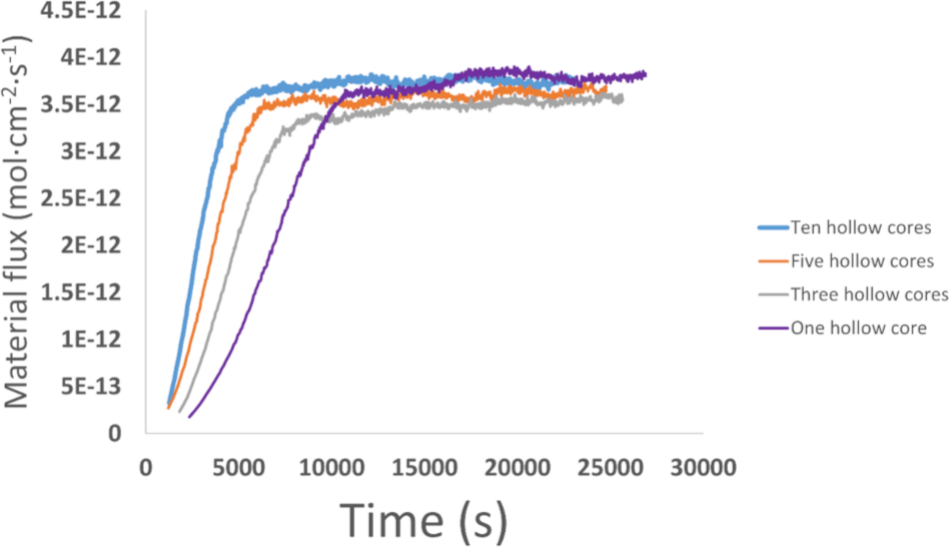
Material flux vs time for systems with different densities of hollow cores. Each trajectory on the plot represents the average of ten runs with a fixed dislocation density but different dislocation locations.

The primary effect of dislocation density on the material flux is the time it takes for the system to reach a steady state, a phenomenon also observed by Lasaga and Blum^69^ for quartz dissolution. Kurganskaya and Luttge^102^ found that the stochastic change in the dissolution regime overrides the number of dislocations, resulting in a uniform dissolution rate across various dislocation densities. The effect of dislocation plays a significant role in the dissolution process only for cases with extremely high dislocation densities, which occur in highly strained nano- or micrograins. The effect of the dislocation density may be significantly higher when considering a broader range of densities (for example, 10^5^–10^10^ cm^–2^), which is common in natural minerals. In our case, we are limited by the system size that can be modeled in our kMC simulations.

In general, we observed that the increase in the hollow core density positively affects the time it takes for the system to reach a steady state. However, when the dislocation density reaches 5 × 10^7^, most of the hollow cores do not contribute to the dissolution process due to their overlap with more dominant hollow cores. The character of the material flux oscillations is dependent on the relative positions and interactions of the etch pits.

#### Change in Material Flux with Temperature

Information on the change in the material flux at elevated temperatures is critical for nuclear safety engineers. The nuclear waste in repositories is continuously heated due to the nuclear decay of radioactive isotopes.

We investigated the effect of temperature on material flux by running a series of simulations on systems with ten randomly distributed hollow cores at temperatures of 22, 55, and 90 °C. The relationship between material flux and temperature is described by the Arrhenius equation (eq 9). The value of the material flux increases exponentially with temperature.

The material flux reached steady-state in 5 × 10^3^, 250, and 30 s and reached the approximate values of 3.5 × 10^–12^ ± 3.3 × 10^–13^, 6.0 × 10^–11^ ± 7.0 × 10^–12^, and 7.0 × 10^–10^ ± 6.3^–11^ mol·cm^–2^·s^–1^ at 22, 55, and 90 °C, respectively.

Our data on material flux values differ significantly from those reported in the literature. The first reason for this is that the reported values of material flux were obtained in the experiments at more acidic pH levels. The second reason is that an alternative approach was employed for surface normalization. The third reason for this inconsistency is that fFar-from-equilibrium conditions are difficult to obtain, as experimental solutions can quickly become saturated, which slows the reaction rates. Our data obtained at 22 °C matches within the order of magnitude the data of Zhen Wu and coauthors^121^ obtained at 25 °C by normalization on the geometric surface area. The value of the material flux is slightly higher due to the higher temperature and more acidic pH levels. Despite the significant variance of our modeling conditions from the experimental conditions presented in the literature, we include our data here for the benefit of future investigations (Table 3).

**Table 3:** Material flux values for Barite dissolution in pure water at different conditions

*T* °C	pH	material flux (mol·cm^–2^·s^–1^)	surface calculation	reference
22	7.0	3.7 × 10^–12^	planar system size	this study
25	5.97	5.0 × 10^–12^	geometric surface area	^121^
25	5.97	9.0 × 10^–13^	BET	^121^
40	5.7	3.6 × 10^–13^	total surface area	^122^
42	4.0	9.1 × 10^–13^	total surface area	^122^
50	2.0	1.3 × 10^–11^	total surface area	^122^
50	3.0	6.6 × 10^–12^	total surface area	^122^
50	4.0	4.9 × 10^–12^	total surface area	^122^
50	5.7	3.4 × 10^–12^	total surface area	^122^
55	7.0	6.5 × 10^–11^	planar system size	this study
90	5.7	5.4 × 10^–13^	total surface area	^122^
90	7.0	7.5 × 10^–10^	planar system size	this study

## Conclusions

In this study, we introduced three new concepts based on the provided observations and the results. The most important finding is the nonlinear behavior of material fluxes. The quasi-periodic oscillations of material flux presumably indicate that the studied system may behave according to some quasi-deterministic internal self-inducing mechanism with a stochastic component. The deterministic features of the system may be attributed to the specific properties of the barite crystal structure and its system-specific interactions with stochastic fluctuations. It raises the question of whether the temporal dynamics of dissolution fluxes for certain crystal structures can be predicted in principle, in contrast to systems with largely stochastic behavior, such as calcium carbonate. This issue will be the subject of our further studies.

The two other concepts are related to the parametrization of kMC models. One concept relates the activation energies of bond breaking to the bond length. We have found that implicit consideration of Coulomb interactions by introducing bond lengths’ related parameters improves the parametrization of kMC models for systems with ionic bonding. We suggest that incorporating the effect of bond lengths can be beneficial for kMC models, simulating the dissolution of crystals with highly asymmetric structures. Since testing the applicability of this approach requires the development of a system-specific kMC model, we leave this task to interested researchers. The other concept is related to the fitting of the model to the experimental data at elevated temperatures. This approach allowed us to unambiguously distinguish the effects of the activation barrier and pre-exponential factor, thus mathematically separating these two variables.

We suggest that the new data and theoretical concepts provided in this study can be beneficial for understanding dissolution mechanisms of ionic solids in general and barite, specifically. If our observation on quasi-deterministic behavior can be extrapolated to macroscopic systems (which should be a subject of separate studies), then barite can be considered not only as an efficient material for Ra and Sr incorporation in nuclear waste repository fields but also a material with well-predictable kinetic behavior.

## Supplementary Material



## Notes

Average trajectory of the [010]-slow step rate and the ranges of standard deviation in the monolayer etch pit at 22°C; behavior of the standard deviation of the [010]-slow step rate in the monolayer etch pit at 22°C; average trajectory of the material flux and the ranges of standard deviation in the system with one hollow core; average trajectory of the material flux and the ranges of standard deviation in the system with three hollow cores; average trajectory of the material flux and the ranges of standard deviation in the system with five hollow cores; average trajectory of the material flux and the ranges of standard deviation in the system with ten hollow cores; average trajectory of the material flux and the ranges of standard deviation in the system with 10 hollow cores at 22°C; average trajectory of the material flux and the ranges of standard deviation in the system with ten hollow cores at 55°C; and average trajectory of the material flux and the ranges of standard deviation in the system with ten hollow cores at 90°C (PDF)

## References

[1] Hanor J. S. (2000). Barite–Celestine
Geochemistry and Environments of Formation. Reviews in Mineralogy and Geochemistry.

[2] Paige C. R., Kornicker W. A., Hileman A., Snodgrass W. J. (1998). Solution
Equilibria for Uranium Ore Processing: The BaSO4-H2SO4-H2O System
and the RaSO4-H2SO4-H2O System. Geochim. Cosmochim.
Acta.

[3] Bosbach D., Hall C., Putnis A. (1998). Mineral Precipitation
and Dissolution
in Aqueous Solution: In-Situ Microscopic Observations on Barite (001)
with Atomic Force Microscopy. Chem. Geol..

[4] Dunn K., Yen T. F. (1999). Dissolution of Barium Sulfate Scale
Deposits by Chelating
Agents. Environ. Sci. Technol..

[5] Wang K.-S., Resch R., Dunn K., Shuler P., Tang Y., Koel B. E., Fu Yen T. (1999). Dissolution
of the Barite (001) Surface
by the Chelating Agent DTPA as Studied with Non-Contact Atomic Force
Microscopy. Colloids Surf., A.

[6] Lakatos, I. ; Lakatos-Szabó, J. ; Kosztin, B. Optimization of Barite Dissolvers by Organic Acids and pH Regulation. SPE International Oilfield Scale Conference and Exhibition; SPE 2002, 10.2118/74667-MS.

[7] Jones F., Jones P., Ogden M., Richmond W. R., Rohl A. L., Saunders M. (2007). The Interaction of EDTA with Barium Sulfate. J. Colloid Interface Sci..

[8] Putnis C. V., Kowacz M., Putnis A. (2008). The Mechanism
and Kinetics of DTPA-Promoted
Dissolution of Barite. Appl. Geochem..

[9] Kowacz M., Putnis C. V., Putnis A. (2009). The Control of Solution Composition
on Ligand-Promoted Dissolution: DTPA–Barite Interactions. Cryst. Growth Des..

[10] Ouyang B., Renock D., Akob D. M. (2019). Effects
of Organic Ligands and Background
Electrolytes on Barite Dissolution. Geochim.
Cosmochim. Acta.

[11] Curti E., Fujiwara K., Iijima K., Tits J., Cuesta C., Kitamura A., Glaus M. A., Müller W. (2010). Radium Uptake
during Barite Recrystallization at 23 ± 2 °C as a Function
of Solution Composition: An Experimental 133Ba and 226Ra Tracer Study. Geochim. Cosmochim. Acta.

[12] Vinograd V. L., Brandt F., Rozov K., Klinkenberg M., Refson K., Winkler B., Bosbach D. (2013). WInkler B.; Bosbach
D. Solid–Aqueous Equilibrium in the BaSO4–RaSO4–H2O
System: First-Principles Calculations and a Thermodynamic Assessment. Geochim. Cosmochim. Acta.

[13] Klinkenberg M., Brandt F., Breuer U., Bosbach D. (2014). Uptake of
Ra during
the Recrystallization of Barite: A Microscopic and Time of Flight-Secondary
Ion Mass Spectrometry Study. Environ. Sci. Technol..

[14] Brandt F., Curti E., Klinkenberg M., Rozov K., Bosbach D. (2015). Replacement
of Barite by a (Ba,Ra)­SO4 Solid Solution at Close-to-Equilibrium Conditions:
A Combined Experimental and Theoretical Study. Geochim. Cosmochim. Acta.

[15] Alpers, C. N. ; Jambor, J. L. ; Nordstrom, D. Sulfate Minerals: Crystallography, Geochemistry, and Environmental Significance; Walter de Gruyter GmbH & Co KG, 2018.

[16] Long-term safety for the final repository for spent nuclear fuel at Forsmark. Main report of the SR-Site project. *Updated 2015–05 – SKB.com*. https://www.skb.com/publication/2345580/ (accessed 2025–02–11).

[17] Guembou
Shouop C. J., Bak S.-I., Mekontso E. J. N., Ndontchueng
Moyo M., Strivay D. (2022). Barite Concrete-Based Cement Composites for 252Cf Spontaneous
Neutron and 60Co/192Ir Shielding Based on Monte Carlo Computation. Mater. Res. Express.

[18] Kanagaraj B., Anand N., Andrushia A. D., Naser M. Z. (2023). Recent Developments
of Radiation Shielding Concrete in Nuclear and Radioactive Waste Storage
Facilities – A State of the Art Review. Constr. Build. Mater..

[19] Kinnunen P., Pelto J., Viitanen P., Olin M., Nieminen M. (2024). Valorisation
of Baryte Tailings for Radiation Shielding in Plastics and Nuclear
Waste Disposal. Heliyon.

[20] Chemical Thermodynamics of Solid Solutions of Interest in Nuclear Waste Management. Nuclear Energy Agency (NEA). https://www.oecd-nea.org/jcms/pl_14272/chemical-thermodynamics-of-solid-solutions-of-interest-in-nuclear-waste-management?details=true (accessed 2025–07–21).

[21] Dove P. M., Platt F. M. (1996). Compatible Real-Time Rates of Mineral Dissolution by
Atomic Force Microscopy (AFM). Chem. Geol..

[22] Gasharova B., Gottlicher J., Becker U. (2005). Dissolution at the Surface of Jarosite:
An in Situ AFM Study. Chem. Geol..

[23] Hillner P. E., Manne S., Gratz A. J., Hansma P. K. (1992). AFM Images of Dissolution
and Growth on a Calcite Crystal. Ultramicroscopy.

[24] Kowacz M., Putnis A. (2008). The Effect of Specific
Background Electrolytes on Water
Structure and Solute Hydration: Consequences for Crystal Dissolution
and Growth. Geochim. Cosmochim. Acta.

[25] Kuwahara Y. (2011). In Situ Atomic
Force Microscopy Study of Dissolution of the Barite (001) Surface
in Water at 30°C. Geochim. Cosmochim. Acta.

[26] Pina C. M., Bosbach D., Prieto M., Putnis A. (1998). Microtopography of
the Barite (0 0 1) Face during Growth:: AFM Observations and PBC Theory. J. Cryst. Growth.

[27] Putnis A., Junta-Rosso J. L., Hochella M. F. (1995). Dissolution of
Barite by a Chelating Ligand: An Atomic Force Microscopy Study. Geochim. Cosmochim. Acta.

[28] Shiraki R., Rock P. A., Casey W. H. (2000). Dissolution
Kinetics of Calcite in
0.1 M NaCl Solution at Room Temperature: An Atomic Force Microscopic
(AFM) Study. Aquatic Geochemistry.

[29] Wang L., Putnis C. V. (2020). Dissolution and
Precipitation Dynamics at Environmental
Mineral Interfaces Imaged by In Situ Atomic Force Microscopy. Acc. Chem. Res..

[30] Arvidson R. S., Collier M., Davis K. J., Vinson M. D., Amonette J. E., Luttge A. (2006). Magnesium Inhibition
of Calcite Dissolution Kinetics. Geochim. Cosmochim.
Acta.

[31] Arvidson R. S., Beig M. S., Luttge A. (2004). Single-Crystal Plagioclase
Feldspar
Dissolution Rates Measured by Vertical Scanning Interferometry. Am. Mineral..

[32] Arvidson R. S., Ertan I. E., Amonette J. E., Luttge A. (2003). Variation in Calcite
Dissolution Rates:: A Fundamental Problem?. Geochim. Cosmochim. Acta.

[33] Arvidson R. S., Luttge A. (2010). Mineral Dissolution
Kinetics as a Function of Distance
from Equilibrium – New Experimental Results. Chem. Geol..

[34] Green E., Luttge A. (2006). Incongruent Dissolution
of Wollastonite Measured with
Vertical Scanning Interferometry. Am. Mineral..

[35] Kumar A., Reed J., Sant G. (2013). Vertical Scanning Interferometry:
A New Method to Measure the Dissolution Dynamics of Cementitious Minerals. J. Am. Ceram. Soc..

[36] Lttge A., Winkler U., Lasaga A. C. (2003). Interferometric
Study of the Dolomite
Dissolution: A New Conceptual Model for Mineral Dissolution. Geochim. Cosmochim. Acta.

[37] Satoh, H. ; Ishii, T. ; Owada, H. Dissolution of Compacted Montmorillonite at Hyperalkaline pH and 70°C: In Situ VSI and Ex Situ AFM Measurements. Clay Minerals 2013, 48 (2), 285–294. 10.1180/claymin.2013.048.2.10.

[38] Trindade
Pedrosa E., Kurganskaya I., Fischer C., Luttge A. (2019). A Statistical
Approach for Analysis of Dissolution Rates Including Surface Morphology. Minerals.

[39] Fischer C., Arvidson R. S., Lüttge A. (2012). How Predictable Are Dissolution Rates
of Crystalline Material?. Geochim. Cosmochim.
Acta.

[40] Luttge A., Arvidson R. S. (2010). Reactions at Surfaces: A New Approach Integrating Interferometry
and Kinetic Simulations. J. Am. Ceram. Soc..

[41] Greathouse J. A., Cygan R. T. (2005). Molecular Dynamics Simulation of Uranyl­(vi) Adsorption
Equilibria onto an External Montmorillonite Surface. Phys. Chem. Chem. Phys..

[42] Aryanpour M., van Duin A. C. T., Kubicki J. D. (2010). Development of a Reactive Force Field
for Iron–Oxyhydroxide Systems. J. Phys.
Chem. A.

[43] Cygan R. T., Romanov V. N., Myshakin E. M. (2012). Molecular Simulation of Carbon Dioxide
Capture by Montmorillonite Using an Accurate and Flexible Force Field. J. Phys. Chem. C.

[44] Cygan, R. T. ; Liang, J. J. ; Kalinichev, A. G. Molecular Models of Hydroxide, Oxyhydroxide, and Clay Phases and the Development of a General Force Field *|* J. Phys. Chem. B https://pubs.acs.org/doi/full/10.1021/jp0363287 (accessed 2025–02–06).

[45] Garrison B. J., Kodali P. B. S., Srivastava D. (1996). Modeling of Surface Processes as
Exemplified by Hydrocarbon Reactions. Chem.
Rev..

[46] Garrison B. J., Dawnkaski E. J., Srivastava D., Brenner D. W. (1992). Molecular Dynamics
Simulations of Dimer Opening on a Diamond {001}(2 × 1) Surface. Science.

[47] Greathouse J. A., Cygan R. T. (2006). Water Structure
and Aqueous Uranyl­(VI) Adsorption Equilibria
onto External Surfaces of Beidellite, Montmorillonite, and Pyrophyllite:
Results from Molecular Simulations. Environ.
Sci. Technol..

[48] Ho T. A., Criscenti L. J. (2021). Molecular-Level Understanding of Gibbsite Particle
Aggregation in Water. J. Colloid Interface Sci..

[49] Kalinichev A. G., Wang J., Kirkpatrick R. J. (2007). Molecular
Dynamics Modeling of the
Structure, Dynamics and Energetics of Mineral–Water Interfaces:
Application to Cement Materials. Cem. Concr.
Res..

[50] Kalinichev A. G., Kirkpatrick R. J. (2002). Molecular
Dynamics Modeling of Chloride Binding to
the Surfaces of Calcium Hydroxide, Hydrated Calcium Aluminate, and
Calcium Silicate Phases. Chem. Mater..

[51] Leung K., Criscenti L. J. (2012). Predicting
the Acidity Constant of a Goethite Hydroxyl
Group from First Principles. J. Phys.: Condens.
Matter.

[52] Rimsza J. M., Jones R. E., Criscenti L. J. (2018). Interaction
of NaOH Solutions with
Silica Surfaces. J. Colloid Interface Sci..

[53] Cygan R. T., Greathouse J. A., Heinz H., Kalinichev A. G. (2009). Molecular
Models and Simulations of Layered Materials. J. Mater. Chem..

[54] Vasconcelos I. F., Bunker B. A., Cygan R. T. (2007). Molecular Dynamics Modeling of Ion
Adsorption to the Basal Surfaces of Kaolinite. J. Phys. Chem. C.

[55] Wang J., Kalinichev A. G., Kirkpatrick R. J., Cygan R. T. (2005). Structure, Energetics,
and Dynamics of Water Adsorbed on the Muscovite (001) Surface: A Molecular
Dynamics Simulation. J. Phys. Chem. B.

[56] Wang J., Kalinichev A. G., Kirkpatrick R. J. (2006). Effects of Substrate Structure and
Composition on the Structure, Dynamics, and Energetics of Water at
Mineral Surfaces: A Molecular Dynamics Modeling Study. Geochim. Cosmochim. Acta.

[57] Criscenti L. J., Kubicki J. D., Brantley S. L. (2006). Silicate
Glass and Mineral Dissolution:
Calculated Reaction Paths and Activation Energies for Hydrolysis of
a Q3 Si by H3O+ Using Ab Initio Methods. J.
Phys. Chem. A.

[58] Morrow C. P., Nangia S., Garrison B. J. (2009). Ab Initio Investigation of Dissolution
Mechanisms in Aluminosilicate Minerals. J. Phys.
Chem. A.

[59] Nangia S., Garrison B. J. (2009). Ab Initio Study of Dissolution and Precipitation Reactions
from the Edge, Kink, and Terrace Sites of Quartz as a Function of
pH. Mol. Phys..

[60] Nangia S., Garrison B. J. (2008). Reaction Rates and
Dissolution Mechanisms of Quartz
as a Function of pH. J. Phys. Chem. A.

[61] Ockwig N.
W., Cygan R. T., Criscenti L. J., Nenoff T. M. (2008). Molecular Dynamics
Studies of Nanoconfined Water in Clinoptilolite and Heulandite Zeolites. Phys. Chem. Chem. Phys..

[62] Zapol P., He H., Kwon K. D., Criscenti L. J. (2013). First-Principles Study of Hydrolysis
Reaction Barriers in a Sodium Borosilicate Glass. International Journal of Applied Glass Science.

[63] Zhang Z., Fenter P., Cheng L., Sturchio N. C., Bedzyk M. J., Předota M., Bandura A., Kubicki J. D., Lvov S. N., Cummings P. T., Chialvo A. A., Ridley M. K., Bénézeth P., Anovitz L., Palmer D. A., Machesky M. L., Wesolowski D. J. (2004). Ion Adsorption
at the Rutile–Water Interface: Linking Molecular and Macroscopic
Properties. Langmuir.

[64] Rudin S., Kowalski P. M., Klinkenberg M., Bornhake T., Bosbach D., Brandt F. (2024). Simulation of Crystal
Growth by an Innovative Hybrid
Density Functional Theory Continuum Solvation Approach: Kink Site
Formation on Barite (001). Cryst. Growth Des..

[65] Rudin, S. ; Kowalsky, P. M. ; Klingenberg, M. ; Bosbach, D. ; Brandt, F. Anisotropy of Barite during Crystal Growth and the Uptake of Radium; Crystal Growth & Design. https://pubs.acs.org/doi/full/10.1021/acs.cgd.4c00416 (accessed 2025–02–10).

[66] Stack A. G. (2009). Molecular
Dynamics Simulations of Solvation and Kink Site Formation at the {001}
Barite–Water Interface. J. Phys. Chem.
C.

[67] Stack A. G., Raiteri P., Gale J. D. (2012). Accurate Rates of the Complex Mechanisms
for Growth and Dissolution of Minerals Using a Combination of Rare-Event
Theories. J. Am. Chem. Soc..

[68] Gillespie D. T. (1976). A General
Method for Numerically Simulating the Stochastic Time Evolution of
Coupled Chemical Reactions. J. Comput. Phys..

[69] Lasaga A. C., Blum A. E. (1986). Surface Chemistry, Etch Pits and Mineral-Water Reactions. Geochim. Cosmochim. Acta.

[70] Lasaga A. C., Luttge A. (2001). Variation of Crystal
Dissolution Rate Based on a Dissolution
Stepwave Model. Science.

[71] Voter, A. F. INTRODUCTION TO THE KINETIC MONTE CARLO METHOD. In Radiation Effects in Solids; Sickafus, K. E. ; Kotomin, E. A. ; Uberuaga, B. P. , Eds.; NATO Science Series; Springer Netherlands: Dordrecht, 2007; pp 1–23. 10.1007/978-1-4020-5295-8_1.

[72] Wehrli B. (1989). Monte Carlo
Simulations of Surface Morphologies during Mineral Dissolution. J. Colloid Interface Sci..

[73] Chen J. C., Reischl B., Spijker P., Holmberg N., Laasonen K., Foster A. S. (2014). Ab Initio Kinetic
Monte Carlo Simulations of Dissolution
at the NaCl–Water Interface. Phys. Chem.
Chem. Phys..

[74] Han Y., Liu D.-J., Evans J. W. (2014). Real-Time
Ab Initio KMC Simulation
of the Self-Assembly and Sintering of Bimetallic Epitaxial Nanoclusters:
Au + Ag on Ag(100). Nano Lett..

[75] Jiang C., Aagesen L. K., Andersson D., Matthews C., Badry F. (2021). Bulk and Surface
Diffusion of Neodymium in Alpha-Uranium: Ab Initio Calculations and
Kinetic Monte Carlo Simulations. J. Nucl. Mater..

[76] Martin P., Gaitero J. J., Dolado J. S., Manzano H. (2021). New Kinetic Monte Carlo
Model to Study the Dissolution of Quartz. ACS
Earth Space Chem..

[77] Moon J., Lee B., Cho M., Cho K. (2014). Ab Initio and Kinetic Monte Carlo
Simulation Study of Lithiation in Crystalline and Amorphous Silicon. J. Power Sources.

[78] Sadigh B., Lenosky T. J., Theiss S. K., Caturla M.-J., Diaz
de la Rubia T., Foad M. A. (1999). Mechanism of Boron Diffusion in Silicon:
An Ab Initio and Kinetic Monte Carlo Study. Phys. Rev. Lett..

[79] Xu L., Henkelman G. (2008). Adaptive Kinetic
Monte Carlo for First-Principles Accelerated
Dynamics. J. Chem. Phys..

[80] Zhong K., Yang Y., Xu G., Zhang J.-M., Huang Z. (2017). An Ab Initio
and Kinetic Monte Carlo Simulation Study of Lithium Ion Diffusion
on Graphene. Materials.

[81] Huang W., Bai X. M. (2023). Machine Learning
Based On-the-Fly Kinetic Monte Carlo
Simulations of Sluggish Diffusion in Ni-Fe Concentrated Alloys. J. Alloys Compd..

[82] Kimari J., Jansson V., Vigonski S., Baibuz E., Domingos R., Zadin V., Djurabekova F. (2020). Application
of Artificial Neural
Networks for Rigid Lattice Kinetic Monte Carlo Studies of Cu Surface
Diffusion. Comput. Mater. Sci..

[83] Messina L., Castin N., Domain C., Olsson P. (2017). Introducing Ab Initio
Based Neural Networks for Transition-Rate Prediction in Kinetic Monte
Carlo Simulations. Phys. Rev. B.

[84] Kurganskaya I., Churakov S. V. (2018). Carbonate Dissolution
Mechanisms in the Presence of
Electrolytes Revealed by Grand Canonical and Kinetic Monte Carlo Modeling. J. Phys. Chem. C.

[85] Kurganskaya I., Luttge A. (2016). Kinetic Monte Carlo
Approach To Study Carbonate Dissolution. J.
Phys. Chem. C.

[86] Kurganskaya I., Luttge A. (2013). A Comprehensive Stochastic
Model of Phyllosilicate
Dissolution: Structure and Kinematics of Etch Pits Formed on Muscovite
Basal Face. Geochim. Cosmochim. Acta.

[87] Kurganskaya I., Luttge A. (2013). Kinetic Monte Carlo
Simulations of Silicate Dissolution:
Model Complexity and Parametrization. J. Phys.
Chem. C.

[88] Kurganskaya I., Trofimov N., Luttge A. (2022). A Kinetic Monte Carlo Approach to
Model Barite Dissolution: The Role of Reactive Site Geometry. Minerals.

[89] Colville A. A., Staudhammer K. (1967). A Refinement of the Structure of Barite. Am. Mineral..

[90] Humphrey W., Dalke A., Schulten K. (1996). VMD: Visual Molecular
Dynamics. J. Mol. Graphics.

[91] Momma K., Izumi F. (2011). VESTA 3 for Three-Dimensional
Visualization of Crystal, Volumetric
and Morphology Data. J. Appl. Crystallogr..

[92] Hanwell M. D., Curtis D. E., Lonie D. C., Vandermeersch T., Zurek E., Hutchison G. R. (2012). Avogadro: An Advanced Semantic Chemical
Editor, Visualization, and Analysis Platform. Journal of Cheminformatics.

[93] Bortz A. B., Kalos M. H., Lebowitz J. L. (1975). A New Algorithm for Monte Carlo Simulation
of Ising Spin Systems. J. Comput. Phys..

[94] Kuwahara Y. (2012). In Situ Hot-Stage
AFM Study of the Dissolution of the Barite (001) Surface in Water
at 30–55 °C. Am. Mineral..

[95] Risthaus P., Bosbach D., Becker U., Putnis A. (2001). Barite Scale Formation
and Dissolution at High Ionic Strength Studied with Atomic Force Microscopy. Colloids Surf., A.

[96] Pelmenschikov A., Strandh H., Pettersson L. G. M., Leszczynski J. (2000). Lattice Resistance
to Hydrolysis of Si–O–Si Bonds of Silicate Minerals:
Ab Initio Calculations of a Single Water Attack onto the (001) and
(111) β-Cristobalite Surfaces. J. Phys.
Chem. B.

[97] Higgins S. R., Jordan G., Eggleston C. M., Knauss K. G. (1998). Dissolution Kinetics
of the Barium Sulfate (001) Surface by Hydrothermal Atomic Force Microscopy. Langmuir.

[98] Kurganskaya I., Arvidson R. S., Fischer C., Luttge A. (2012). Does the Stepwave Model
Predict Mica Dissolution Kinetics?. Geochim.
Cosmochim. Acta.

[99] Kuwahara Y., Uehara S., Aoki Y. (1998). Surface Microtopography
of Lath-Shaped
Hydrothermal Illite by Tapping-Mode^TM^ and Contact-Mode
AFM. Clays Clay Miner..

[100] Luttge A. (2006). Crystal Dissolution Kinetics and
Gibbs Free Energy. J. Electron Spectrosc. Relat.
Phenom..

[101] Lasaga A. C., Luttge A. (2003). A Model for Crystal Dissolution. European Journal of Mineralogy.

[102] Kurganskaya I., Luttge A. (2023). Probability Distributions
of Mineral
Dissolution Rates: The Role of Lattice Defects. Front. Water.

[103] Jordan G., Higgins S. R., Eggleston C. M., Knauss K. G., Schmahl W. W. (2001). Dissolution Kinetics of Magnesite
in Acidic Aqueous Solution, a Hydrothermal Atomic Force Microscopy
(HAFM) Study: Step Orientation and Kink Dynamics. Geochim. Cosmochim. Acta.

[104] Vinson M. D., Lüttge A. (2005). Multiple Length-Scale
Kinetics: An
Integrated Study of Calcite Dissolution Rates and Strontium Inhibition. Am. J. Sci..

[105] Fischer C., Kurganskaya I., Schäfer T., Lüttge A. (2014). Variability of Crystal Surface Reactivity: What Do
We Know?. Appl. Geochem..

[106] Jordan G., Rammensee W. (1998). Dissolution
Rates of Calcite (101̅4)
Obtained by Scanning Force Microscopy: Microtopography-Based Dissolution
Kinetics on Surfaces with Anisotropic Step Velocities. Geochim. Cosmochim. Acta.

[107] Holdren G. R., Berner R. A. (1979). Mechanism of Feldspar
Weathering—I. Experimental Studies. Geochimica
et Cosmochimica Acta.

[108] Berner R. A., Holdren G. R. (1979). Mechanism of Feldspar
Weathering—II.
Observations of Feldspars from Soils. Geochim.
Cosmochim. Acta.

[109] Berner R. A., Holdren G. R. (1977). Mechanism of
Feldspar Weathering: Some Observational Evidence. Geology.

[110] Berner R. A., Schott J. (1982). Mechanism of Pyroxene
and Amphibole
Weathering; II, Observations of Soil Grains. Am. J. Sci..

[111] Brantley S. L., Crane S. R., Crerar D. A., Hellmann R., Stallard R. (1986). Dissolution at Dislocation Etch Pits in Quartz. Geochim. Cosmochim. Acta.

[112] Helgeson H. C., Murphy W. M., Aagaard P. (1984). Thermodynamic
and Kinetic
Constraints on Reaction Rates among Minerals and Aqueous Solutions.
II. Rate Constants, Effective Surface Area, and the Hydrolysis of
Feldspar. Geochim. Cosmochim. Acta.

[113] Holdren G. R., Speyer P. M. (1985). pH Dependent Changes in the Rates
and Stoichiometry of Dissolution of an Alkali Feldspar at Room Temperature. Am. J. Sci..

[114] Wilson M. J. (1975). CHEMICAL WEATHERING OF SOME PRIMARY
ROCK-FORMING MINERALS. Soil Science.

[115] Casey W. H., Carr M. J., Graham R. A. (1988). Crystal Defects
and the Dissolution Kinetics of Rutile. Geochim.
Cosmochim. Acta.

[116] Murr L. E., Hiskey J. B. (1981). Kinetic Effects
of Particle-Size
and Crystal Dislocation Density on the Dichromate Leaching of Chalcopyrite. Metall Trans B.

[117] Schott J., Brantley S., Crerar D., Guy C., Borcsik M., Willaime C. (1989). Dissolution Kinetics of Strained
Calcite. Geochim. Cosmochim. Acta.

[118] Holdren G. R., Casey W. H., Westrich H. R., Carr M., Boslough M. (1988). Bulk Dislocation Densities and Dissolution
Rates in
a Calcic Plagioclase. Chem. Geol..

[119] Murphy W. M. (1989). Dislocations and Feldspar Dissolution. European Journal of Mineralogy.

[120] Murphy W. M. (1988). Dislocations and Feldspar Dissolution:
Theory and Experimental
Data. Chem. Geol..

[121] Zhen-Wu B. Y., Dideriksen K., Olsson J., Raahauge P. J., Stipp S. L. S., Oelkers E. H. (2016). Experimental
Determination of Barite
Dissolution and Precipitation Rates as a Function of Temperature and
Aqueous Fluid Composition. Geochim. Cosmochim.
Acta.

[122] Dove P. M., Czank C. A. (1995). Crystal Chemical Controls on the
Dissolution Kinetics of the Isostructural Sulfates: Celestite, Anglesite,
and Barite. Geochim. Cosmochim. Acta.

