# Supplementary material for: Mechanisms of Barium Sulfate Dissolution through the Lens of Kinetic Monte Carlo Simulations
Source: ACS Omega. 2025 Oct 10;10(41):49216–30. doi: 10.1021/acsomega.5c05761 (PMC12547578; doi:10.1021/acsomega.5c05761)
Supplement: Supplementary file 1 [file ao5c05761_si_001.pdf]

Mechanisms of barium sulfate dissolution through the lens of kinetic Monte Carlo simulations

Nikolai Trofimov[1,], Andreas Luttge[1,2], and Inna Kurganskaya[1]*

[1]University of Bremen, FB5 Geo, Klagenfurter Str. 4, 28359, Bremen, Germany

[2]Rice University, Earth, Environmental, and Planetary Sciences Department, 6100 Main St, TX77005, Houston, United States

[*] Corresponding author: trofimov@uni-bremen.de

Supporting information

1. Monolayer step rates

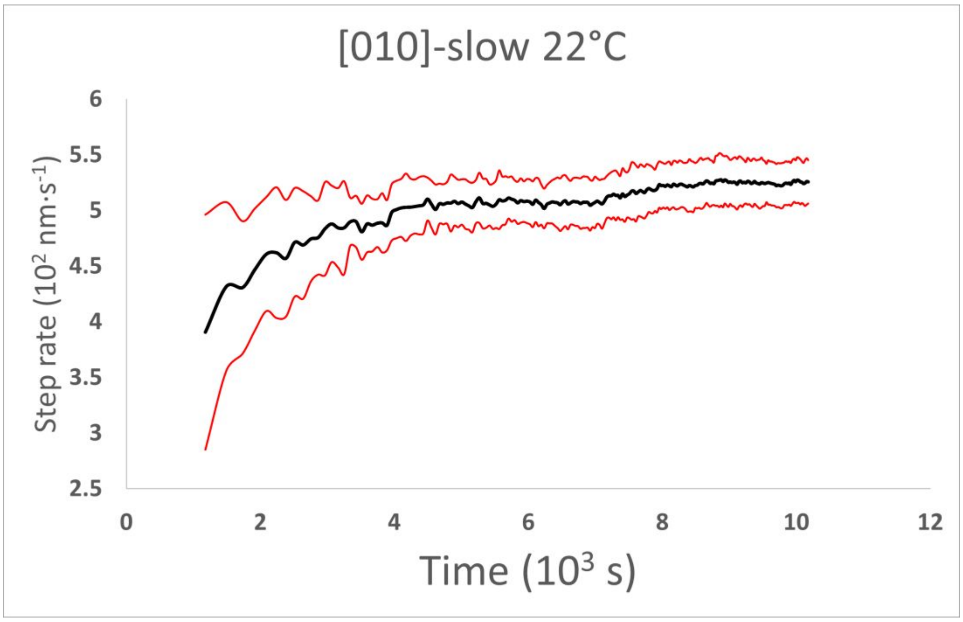

Figure S1. The average trajectory of the [010]-slow step rate (black line) and the ranges of standard deviation (red lines) In the monolayer etch pit at 22°C.

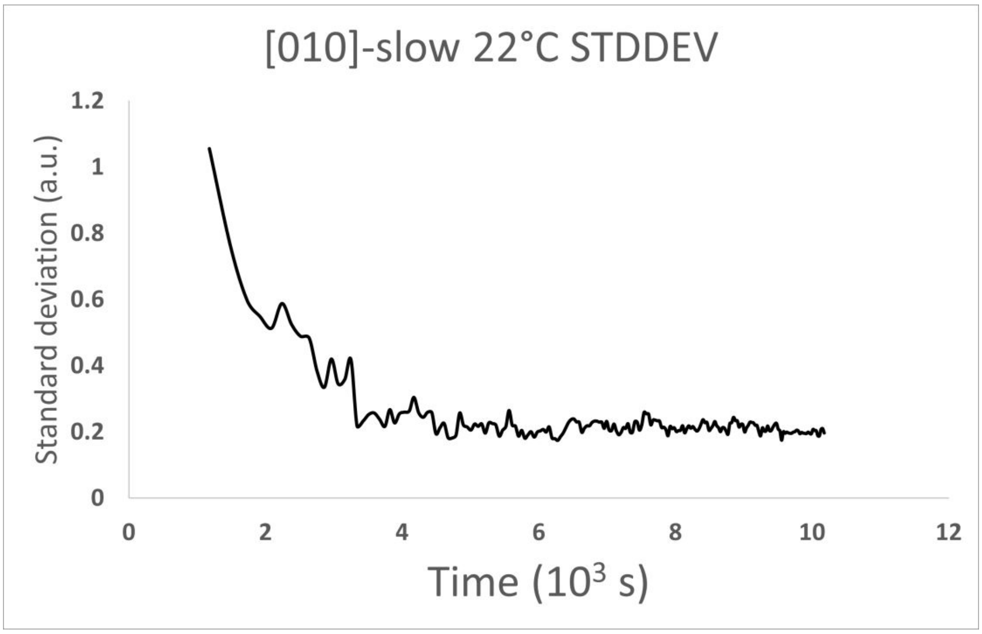

Figure S2. The behavior of the standard deviation of the [010]-slow step rate in the monolayer etch pit at 22°C.

2. Material flux data

Dependence of material flux on dislocation density

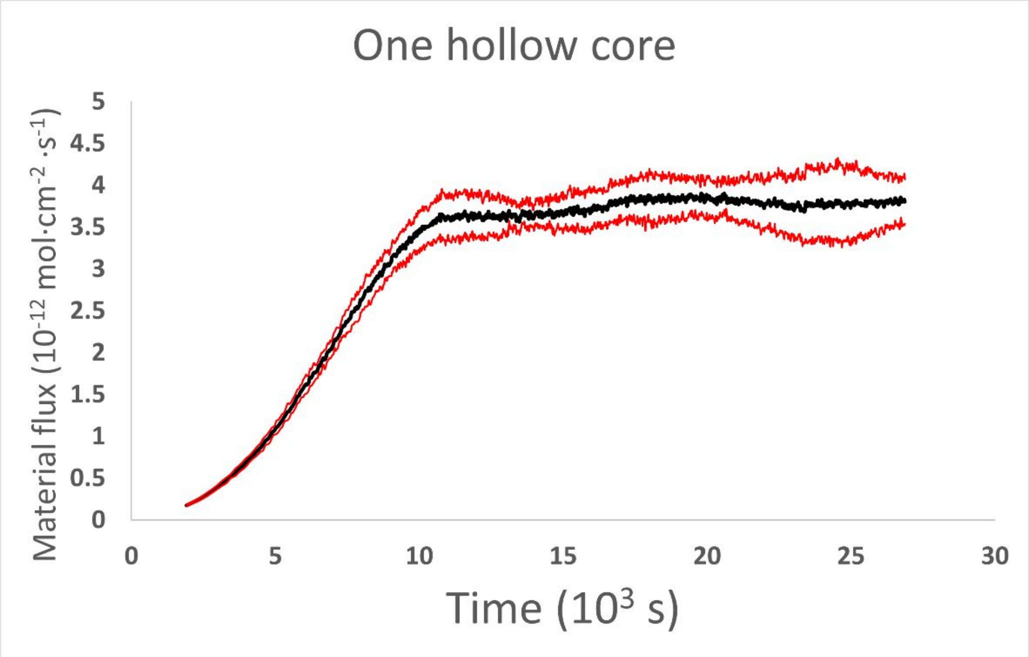

Figure S3. The average trajectory of the material flux (black line) and the ranges of standard deviation (red lines) in the system with one hollow core

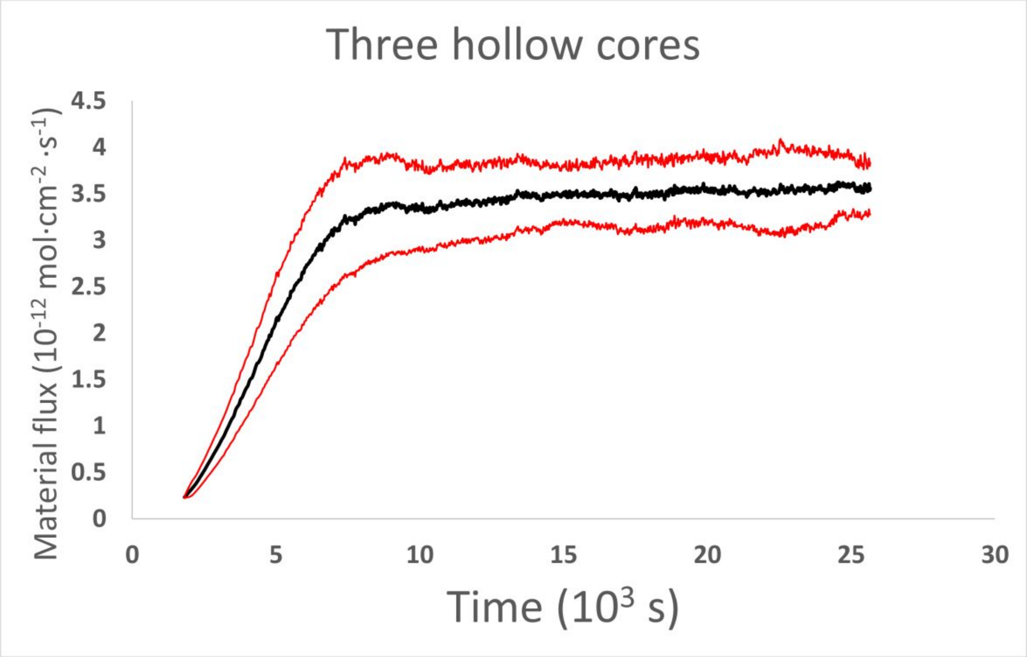

Figure S4. The average trajectory of the material flux (black line) and the ranges of standard deviation (red lines) in the system with three hollow cores

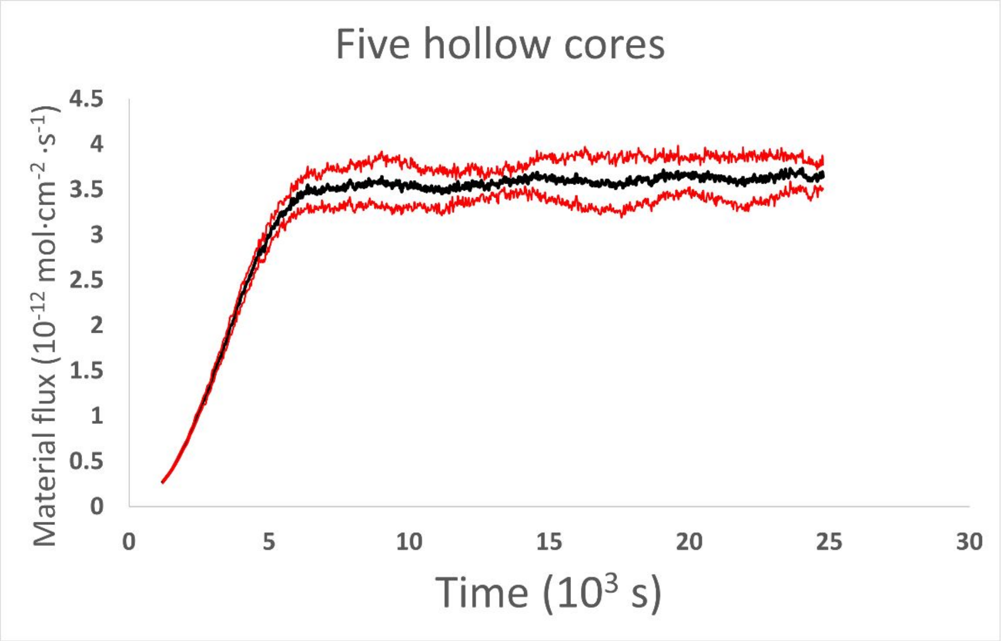

Figure S5. The average trajectory of the material flux (black line) and the ranges of standard deviation (red lines) in the system with five hollow cores

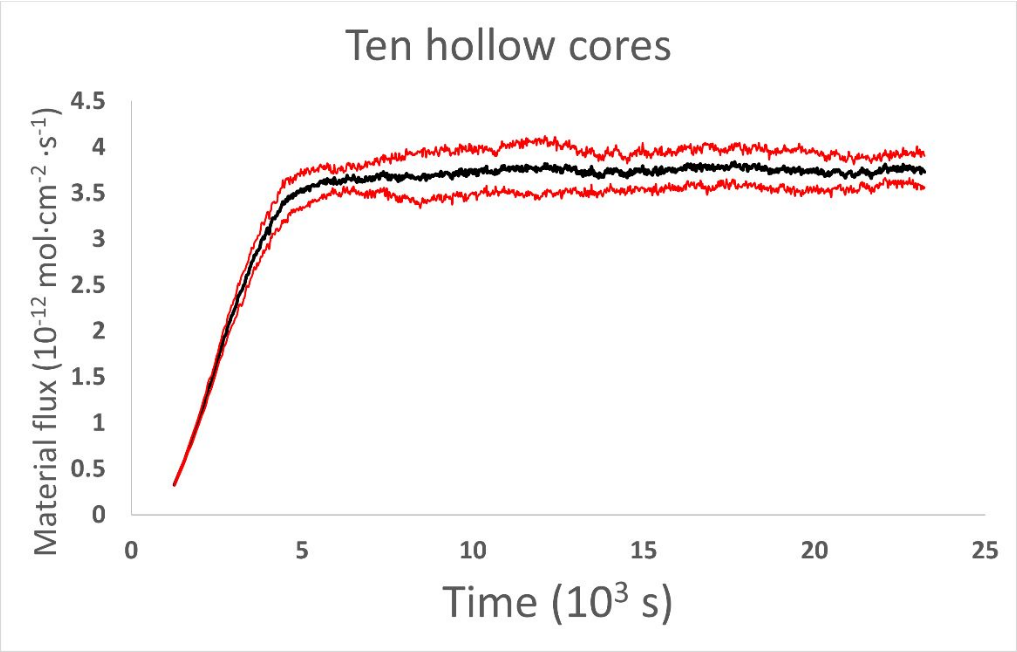

Figure S6. The average trajectory of the material flux (black line) and the ranges of standard deviation (red lines) in the system with ten hollow cores

Dependence of material flux on temperature

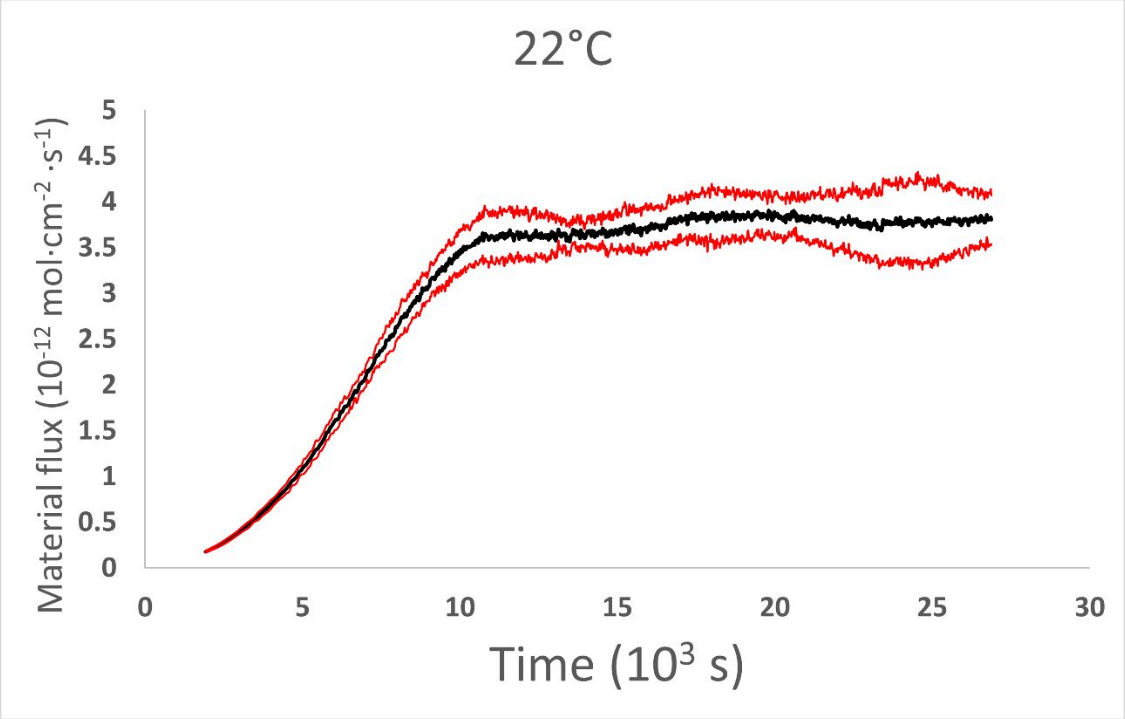

Figure S7. The average trajectory of the material flux (black line) and the ranges of standard deviation (red lines) in the system with ten hollow cores at 22°C

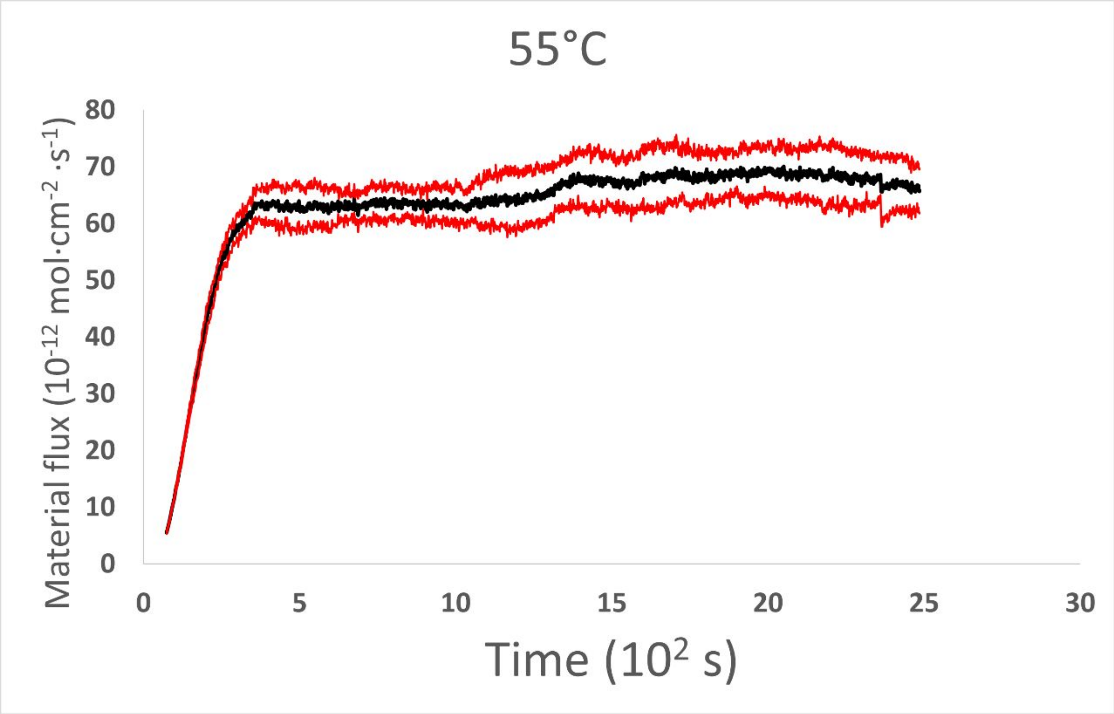

Figure S8. The average trajectory of the material flux (black line) and the ranges of standard deviation (red lines) in the system with ten hollow cores at 55°C

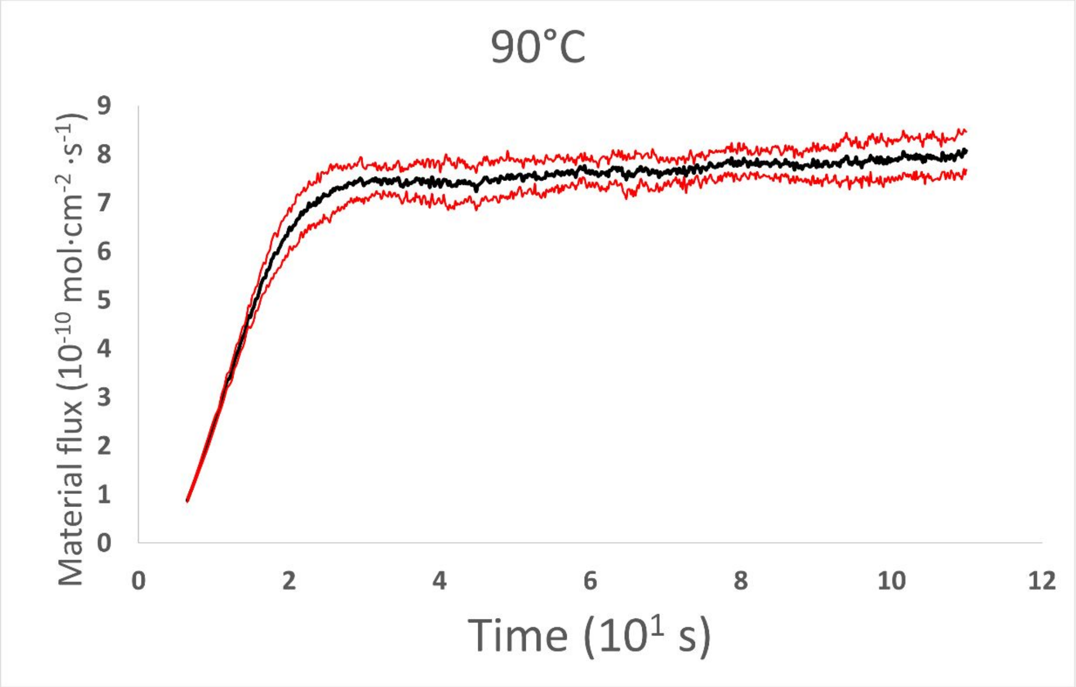

Figure S9. The average trajectory of the material flux (black line) and the ranges of standard deviation (red lines) in the system with ten hollow cores at 90°C
